# Molecular Mechanisms of Alzheimer’s Disease Induced by Amyloid-β and Tau Phosphorylation Along with RhoA Activity: Perspective of RhoA/Rho-Associated Protein Kinase Inhibitors for Neuronal Therapy

**DOI:** 10.3390/cells14020089

**Published:** 2025-01-10

**Authors:** Eun Hee Ahn, Jae-Bong Park

**Affiliations:** 1 Department of Physiology, Hallym University College of Medicine, Chuncheon 24252, Kangwon-do, Republic of Korea; eunhee.ahn@hallym.ac.kr; 2 Department of Neurology, Hallym University College of Medicine, Chuncheon 24252, Kangwon-do, Republic of Korea; 3 Department of Biochemistry, Hallym University College of Medicine, Chuncheon 24252, Kangwon-do, Republic of Korea; 4 Institute of Cell Differentiation and Aging, Hallym University College of Medicine, Chuncheon 24252, Kangwon-do, Republic of Korea; 5 ELMED Co., Hallym University College of Medicine, Chuncheon 24252, Kangwon-do, Republic of Korea

**Keywords:** Alzheimer’s disease, amyloid-beta, tau phosphorylation, neuroinflammation, RhoA GTPase

## Abstract

Amyloid-β peptide (Aβ) is a critical cause of Alzheimer’s disease (AD). It is generated from amyloid precursor protein (APP) through cleavages by β-secretase and γ-secretase. γ-Secretase, which includes presenilin, is regulated by several stimuli. Tau protein has also been identified as a significant factor in AD. In particular, Tau phosphorylation is crucial for neuronal impairment, as phosphorylated Tau detaches from microtubules, leading to the formation of neurofibrillary tangles and the destabilization of the microtubule structure. This instability in microtubules damages axons and dendrites, resulting in neuronal impairment. Notably, Aβ is linked to Tau phosphorylation. Another crucial factor in AD is neuroinflammation, primarily occurring in the microglia. Microglia possess several receptors that bind with Aβ, triggering the expression and release of an inflammatory factor, although their main physiological function is to phagocytose debris and pathogens in the brain. NF-κB activation plays a major role in neuroinflammation. Additionally, the production of reactive oxygen species (ROS) in the microglia contributes to this neuroinflammation. In microglia, superoxide is produced through NADPH oxidase, specifically NOX2. Rho GTPases play an essential role in regulating various cellular processes, including cytoskeletal rearrangement, morphology changes, migration, and transcription. The typical function of Rho GTPases involves regulating actin filament formation. Neurons, with their complex processes and synapse connections, rely on cytoskeletal dynamics for structural support. Other brain cells, such as astrocytes, microglia, and oligodendrocytes, also depend on specific cytoskeletal structures to maintain their unique cellular architectures. Thus, the aberrant regulation of Rho GTPases activity can disrupt actin filaments, leading to altered cell morphology, including changes in neuronal processes and synapses, and potentially contributing to brain diseases such as AD.

## 1. Introduction

**Figure 1:**
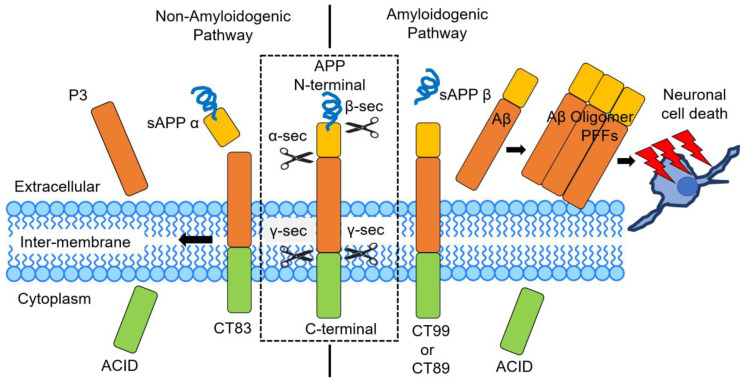
The process of Aβ generation from amyloid precursor protein (APP). The amyloid precursor protein (APP) undergoes cleavage by multiple proteases, including α-secretase, β-secretase, and γ-secretase, resulting in the production of various peptides. Cleavage of APP by β-secretase and γ-secretase specifically generates monomeric, oligomeric, and polymeric aggregates of Aβ40 and Aβ42. Of these, the oligomeric forms of Aβ42 are recognized as the most toxic agents in AD.

Alzheimer’s disease (AD) is the leading cause of dementia, affecting approximately 45 million individuals worldwide [1,2]. Pathological features of AD are typically characterized by the accumulation of amyloid plaques, composed of amyloid-β (Aβ) peptides, and neurofibrillary tangles (NFTs) made up of hyperphosphorylated Tau. Increasing evidence points to Aβ and p-Tau are key biomarkers in AD [3]. Aβ is a major component of senile plaques, with studies identifying thousands of proteins in senile plaques [4]. Breakthroughs in molecular medicine have underscored the central role of the Aβ pathway in AD pathophysiology [5,6,7]. Although the intricate molecular mechanisms and spatial-temporal dynamics leading to synaptic failure, neurodegeneration, and clinical onset are still being intensively studied, the established biochemical changes in the Aβ cycle remain a hallmark of AD [8,9,10]. These alterations present promising targets for developing disease-modifying therapies. Aβ is released from the amyloid precursor protein (APP) through the sequential cleavage of α-, β-, and γ-secretase. In the non-amyloidogenic pathway, APP is primarily cleaved by α-secretase within the Aβ domain at the Aβ17 site, producing a secreted form of APP and an 83-amino acid membrane-bound C-terminal fragment (CTF) called C83, thus preventing Aβ production. The β-site APP cleaving enzyme 1 (BACE1), known as β-secretase, and its homolog BACE2, known as θ-secretase, also play a role in the pre-amyloidogenic molecular pathway [11,12]. Under physiological conditions, BACE1 primarily processes APP at the Aβ Glu11 β-secretase site, generating C89, which is then cleaved by γ-secretase to produce a truncated Aβ11-40. BACE2, in contrast, cleaves APP at the Aβ Phe20 θ-secretase site, generating C80 and preventing Aβ formation. In the amyloidogenic pathway, Aβ production requires two enzymatic cleavages of APP by BACE1 and γ-secretase. First, BACE1 cleaves APP at the Asp1 site, generating sAPPβ and C99. Then, γ-secretase cleaves C99 to release Aβ and the C-terminal fragment CTFγ (Figure 1). γ-Secretase is a macromolecular complex [13,14,15,16] containing presenilin 1 (PS1), requiring nicastrin, anterior pharynx-defective 1 (APH-1), and presenilin enhancer 2 (PEN-2) for its enzymatic activity. Interestingly, Aβ42 can induce the phosphorylation of APP at Thr668 through GSK-3β, CDK5, JNK3, and Dyrk1 [17,18]. When APP is phosphorylated at Thr668, it readily interacts with BACE1, enhancing Aβ production through increased APP cleavage by BACE1 [19]. However, Fe65 protein and PP1γ protein phosphatase interact with APP, facilitating the dephosphorylation of the Thr668 residue of APP [20]. Additionally, the phosphorylation of APP at Tyr687 influences its subcellular localization and decreases its turnover rate [21].

Another hallmark of AD is the presence of NFTs, which are aggregates of hyper-phosphorylated Tau released from microtubules. Microtubules form part of the cytoskeleton, supporting the structural and functional integrity of axons and dendrites in neurons. Tau is a type of microtubule-associated protein (MAP) that stabilizes microtubules [22]. However, when Tau is hyperphosphorylated by Tau kinases, it dissociates from microtubules. The phosphorylation status of Tau is controlled by a balance between Tau kinases and phosphatase activities, with many kinases documented to phosphorylate Tau at different sites [23]. Notably, Aβ can induce Tau phosphorylation through specific kinases such as GSK-3β and CDK5 [24,25,26]. Aβ also modulates Tau through the regulation of protein kinases and protein phosphatases [18]. Mice overexpressing APP mutants exhibit features of familial early-onset AD. However, the loss of Tau genes in these APP mutant mice protects them from learning and memory deficits, as well as the excitotoxicity observed in the parental APP strain [27]. These findings suggest that Aβ initiates a pathway leading to Tau-dependent synaptic dysfunction, positioning Aβ as functioning upstream of Tau [28,29]. In contrast, APP/PS1 mice lacking Tau genes show reduced plaque burdens comparted to age-matched APP/PS1 mice with normal Tau expression, suggesting that Tau may regulate Aβ accumulation [28]. These results imply a potential pathological feedback loop in which Aβ initiates a vicious cycle involving Tau [29].

**Figure 2:**
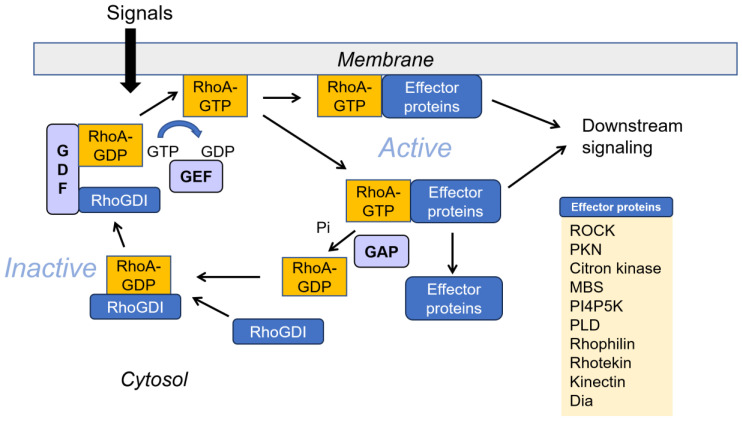
Regulation of RhoA activity by various regulatory proteins. Inactive RhoA, bound to RhoGDI, is dissociated by GDF, enabling RhoA to be activated through GTP incorporation facilitated by GEF. The active RhoA-GTP interacts with various effector proteins, each contributing to specific cellular functions. GAP facilitates the conversion of RhoA-GTP back to RhoA-GDP, terminating its activity. Abbreviations: ROCK, Rho-associated kinase; PKN, protein kinase N; PLD, phospholipase D; MBS, myosin-binding subunit.

Rho GTPases play critical roles in regulating various cellular processes, including cytoskeletal rearrangement, changes of cell morphology, migration and transcription [30]. A key function of Rho GTPases is regulating the formation of actin filaments. In neurons, cytoskeletal dynamics support processes such as the development of axons and dendrites and synapse formation to connect with other neurons. Similarly, brain cells like astrocytes, microglia and oligodendrocytes rely on cytoskeletal structures to develop specific cellular features. Thus, the aberrant regulation of Rho GTPase activity can disrupt actin filament, altering cell morphology, including neuronal processes and synapses, which may contribute to brain diseases such as AD [31]. Rho GTPases, members of the Ras-related small GTP binding protein family—including RhoA, Cdc42 and Rac1/2—are crucial in numerous cellular functions. They play roles in cytoskeletal rearrangement, reactive oxygen species (ROS) production, and the regulation of cell morphology, cell movement, and transcription [30]. The dysregulation of Rho GTPases is associated with various cancer types and neurodegenerative diseases. Rho GTPases are activated when bound to GTP—a process facilitated by guanine nucleotide exchange factors (GEFs). In contrast, GTP hydrolysis, which converts the GTPase back to its inactive GDP-bound state, is promoted by GTPase-activating proteins (GAPs) [32,33,34]. This dynamic cycling between active and inactive states, controlled by specific GEFs and GAPs, is essential for the precise regulation of cellular process. Additionally, Rho GTPases undergo lipid modification by attaching a prenyl group to a cysteine residue in their C-terminal CAAX motif (C for cysteine, A for aliphatic amino acid, and X for any amino acid). In their inactive GDP-bound form, they remain in the cytosol in a complex with RhoGDI (guanine nucleotide dissociation inhibitor). Upon activation, the GTP-bound Rho associates with the cell membrane and is anchored by its prenyl group. For activation, Rho GTPases must be dissociated from RhoGDI, a step enabled by GDI displacement factors (GDFs), as GEF cannot directly act on the Rho GTPase-RhoGDI complex directly [35]. Activated RhoA binding with GTP regulates its function through the interaction with many effector proteins, including Rho-associated protein kinase (ROCK), protein kinase N (PKN), Dia, phospholipase D (PLD), Rhotekin, and Rhophilin [36] (Figure 2)

## 2. The Physiological Functions of APP and Aβ

APP is a ubiquitously expressed protein, and its cleavage generates Aβ peptide fragments. To investigate the physiological function of APP, researchers use genetically altered mice. Mice expressing a truncated form of APP (APPΔ mice) exhibit severe impairments in spatial learning and exploratory behavior [37]. Although homozygous APP-deficient mice are viable and fertile, APP-null mice show reduced locomotor activity, decreased forelimb grip strength, and a high probability of reactive gliosis [38]. Additionally, homozygous APP-deficient mice have impaired performance in the Morris water-maze, indicating defects in spatial memory [37,39], and exhibit difficulties in passive avoidance learning [40]. Furthermore, APP-null mice show a significant loss of presynaptic terminal vesicle marker proteins, such as synaptophysin and synapsin, and the dendritic marker protein MAP2, particularly in the cortex and hippocampus [39]. These findings indicate that APP is essential for normal neuronal functions.

Since Aβ is present in the brains of all healthy individuals, it has been proposed that Aβ also has normal physiological roles in the brain. In mice, picomolar and low nanomolar concentrations of Aβ have been shown to support undifferentiated neurons and to promote long-term potentiation (LTP), a process essential for memory formation. However, higher nanomolar and micromolar concentrations of Aβ are toxic to neurons [41]. Increasing synaptic Aβ, either through direct administration or by inhibiting its degradation, enhances synaptic release and neurotransmission by binding to APP and promoting its dimerization, which results in calcium influx and vesicle release [42]. Conversely, reducing synaptic Aβ levels, either through direct antibody treatment or APP knockout, impairs LTP, suggesting that synaptic Aβ plays a regulatory role in synaptic neurotransmission [43].

In relation to pathogens infecting the brain, Aβ plays a protective role in innate immunity [44]. Soluble Aβ oligomers (AβOs) bind to the microbial cell wall via the heparin-binding domain. Additionally, developing protofibrils interfere with pathogen adhesion to host cells, while propagating Aβ fibrils trigger agglutination, ultimately trapping unattached microbes [45]. AβOs can make pores in invading pathogens, thereby protecting the brain from infection. However, AβOs can also form pores in brain cells, leading to cellular damage [46]. A substantial body of evidence suggests that AβOs are a critical factor in the development of AD through various mechanisms. These observations highlight the concentration-dependent effects of reactive oxygen species (ROS), where both excessively low and too-high levels of ROS impair cellular functions, while an optimal concentration is necessary for normal cellular processes [47].

## 3. Direct Effect of Cell and Membrane Damage by Aβ Through Metal Ion-Induced ROS

Another proposed function of Aβ is to sequester metal ions, such as copper and zinc [41]. High concentrations of copper have been found near Aβ amyloid deposits in AD, often alongside oxidative stress markers. Additionally, Cu^2+^ significantly enhances Aβ-induced neurotoxicity in cell cultures. Notably, the copper-Aβ42 complex can generate H_2_O_2_ as it reduces Cu^2+^ to Cu^1+^. This suggests that redox-active metal ions may play a crucial role in Aβ-mediated oxidative damage in AD [48]. Interestingly, the redox-inactive Zn^2+^ competes with Cu^2+^ for binding to Aβ42, with Zn^2+^ thereby suppressing Aβ42-mediated and Cu^2+^-dependent hydrogen peroxide formation [49]. Interestingly, Zn^2+^ precipitates Aβ in vitro, leading to the proposal that Zn^2+^-induced Aβ plaque formation may have a protective role as an antioxidant response to oxidative damage by Cu^2+^ [49]. However, opposing findings suggest that the Zn^2+^-mediated-aggregation of Aβ peptides—a key pathological event in AD—can be effectively inhibited by Cu^2+^ when competing at a Cu/Aβ molar ratio of 4 [50]. This suggests that ratios among Cu^2+^, Zn^2+^, and Aβ may be critical in determining their beneficial or harmful effects in the brain. Lipid peroxidation has been observed radiating outward from the center of amyloid plaques in the hippocampus [51]. Additionally, inflammatory factors such as IL-1β, IL-6, and TNF-α, as well as copper, significantly reduce the expression of low-density lipoprotein-related protein 1 (LRP1), which facilitates Aβ clearance from the brain [52]. This copper-mediated downregulation of LRP1 is dependent on proteasomal degradation [53].

## 4. Neuronal Damage by Aβ Through Its Receptors

Aβ interacts with various brain cells, including neurons, microglia, astrocytes and oligodendrocytes through multiple receptors on their membrane surfaces [54]. These receptor proteins bind to monomeric, oligomeric, or fibrillar forms of Aβ, with beneficial or harmful effects. Beneficial receptors internalize Aβ for degradation, such as through microglial phagocytosis, or promote its clearance from the brain through transcytosis across endothelial cells. In contrast, harmful receptors bind to oligomeric forms of Aβ that contribute to synaptic loss, memory impairment, and neurotoxicity, as seen in AD [54].

### 4.1. Aβ Receptors in Neurons

#### 4.1.1. Cellular Prion Protein (PrP^C^)

**Figure 3:**
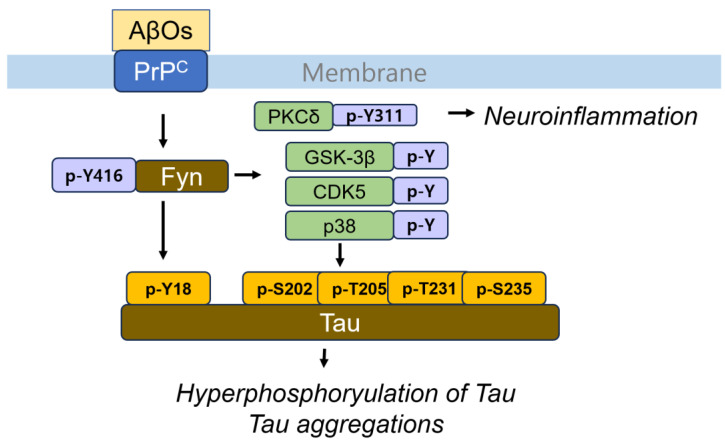
PrP^C^ as a receptor for Aβ in neurons. PrP^C^ binds to Aβ at neuronal dendritic spines, forming a complex with Fyn. This interaction activates Fyn through phosphorylation at Tyr416, resulting in Fyn-dependent hyperphosphorylation of Tau at Tyr18 residue and subsequent phosphorylation at S202/T205 and T231/S235. Fyncan also activates serine/threonine kinases, such as GSK-3β, CDK5, and p38 MAPK, through Fyn-mediated tyrosine phosphorylation. Furthermore, in primary microglia, Fyn phosphorylates PKCδ at the Tyr311 residue, contributing to a neuroinflammatory response.

The cellular prion protein (PrP^C^), but not infectious PrP^Sc^, has been identified as a receptor for AβOs, binding AβOs with high affinity (Kd~4 nM) but not monomers or fibrils [55]. Synaptic responsiveness in young adult PrP^C^-null mice remains normal, and AβOs do not block long-term potentiation in these PrP^C^-deficient mice. Additionally, anti-PrP^C^ antibodies prevent AβO binding to PrP^C^, thereby restoring synaptic plasticity in the hippocampal slice [55]. In familial AD model transgenic mice expressing APP^swe^/PSen^ΔE9^, the deletion of PrP^C^ (Prnp−/−) rescues them from axonal degeneration, the loss of synaptic markers, and early mortality, with no detectable impairments in spatial learning and memory [56]. More specifically, PrP^C^ prevents Aβ fibril polymerization by binding to the rapidly growing ends of each fibril. This interaction blocks polarized elongation, leading to PrP^C^ binding to neurotoxic oligomers and protofibrils, which initiates a neurotoxic signal [57]. Based on these findings, PrP^C^ is proposed to mediate AβOs-induced synaptic dysfunction. In the downstream signaling pathway, PrP^C^ binding with Aβ at the neuronal dendritic spines forms a complex with Fyn, an Src family tyrosine kinase [58]. This interaction activates Fyn through Tyr416 phosphorylation, leading to the Fyn-dependent hyperphosphorylation of Tau at the Tyr18 residue [59]. Interestingly, Fyn also induces the phosphorylation of S202/T205 (detected by AT8 antibody) and T231/S235 (detected by AT180 antibody), resulting in Tau aggregation and the formation of neurofibrillary tangles [60]. Fyn may activate serine/threonine kinases by Fyn-mediated tyrosine phosphorylation, as it is known to phosphorylate GSK-3β, CDK5, and p38 MAPK. Fyn forms a complex with GSK-3β in response to insulin signaling, phosphorylating Tyr residue in GSK-3β and thereby activating it, which subsequently leads to Tau phosphorylation [61] (Figure 3).

**Figure 4:**
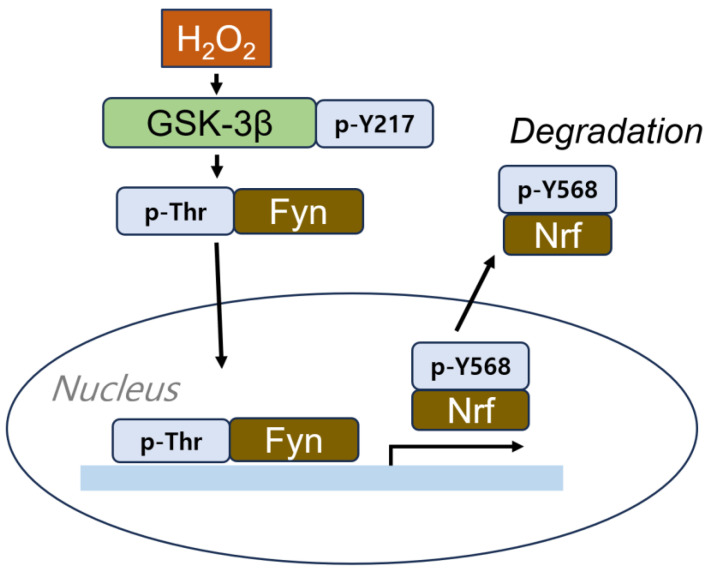
Impairment of Nrf2 in AD. GSK-3β, activated through Tyr216 phosphorylation in response to hydrogen peroxide, promotes the phosphorylation of Fyn at a threonine residue. This phosphorylated Fyn accumulates in the nucleus, where it phosphorylates Nrf2 at Tyr568. This modification leads to the export of Nrf2 from the nucleus and its subsequent degradation.

Conversely, GSK-3β, when activated by Tyr216 phosphorylation in response to hydrogen peroxide, stimulates Fyn phosphorylation at a threonine residue. This phosphorylated Fyn accumulates in the nucleus, where it phosphorylates nuclear factor erythroid 3-related factor 2 (Nrf2) at Tyr568, causing Nrf2 to be exported from the nucleus and degraded [62] (Figure 4). Nrf2 is a critical transcription factor that mitigates oxidative stress by interacting with the antioxidant responsive element (ARE) in the nucleus, thereby promoting the expression of antioxidants and anti-inflammatory genes. The impairment of Nrf is commonly observed in various neurodegenerative diseases, including AD [63]. Semaphorin-3A (Sema3A) induces a growth cone collapse response through binding to its receptor, plexin A. However, this response is attenuated in Fyn- and Cdk5-deficient mice. Sema3A enhances Cdk5 activity through phosphorylation at Tyr15 by Fyn. The active form of the Cdk5 phosphorylated at Tyr15 by Fyn can phosphorylate Tau, leading to microtubule disruption [64]. Additionally, Fyn also phosphorylates PKCδ at the Tyr311 residue in primary microglia, which is relevant to the neuroinflammatory response [65] (Figure 3).

#### 4.1.2. Metabotropic Glutamate Receptor 5 (mGluR5)

**Figure 5:**
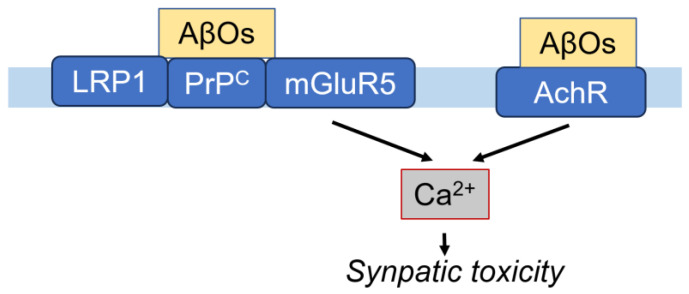
Metabolic glutamate receptor and nicotinic acetylcholine receptor-mediated cytosolic Ca^2+^ elevation as major toxicity-inducing mechanisms in response to Aβ. PrP^C^ interacts with co-receptors LRP1 and mGluR5. AβOs cause abnormal accumulation and overstabilization of mGluR5 at the synapse, leading to elevated intracellular calcium levels and increased synaptic toxicity. Additionally, soluble Aβ also interacts with α7nAchR, resulting in heightened calcium permeability.

Glutamate receptors are divided into two groups: ionotropic receptors, which form ion channel pores, and metabotropic receptors, which affect the cell through signal transduction cascades. The ionotropic receptors include those for NMDA (N-methyl-D-aspartate), a Ca^2+^ ion channel found in neurons [66], as well as kinate and AMPA (α-amino-3-hydroxy-5-methyl-4-isoxasolepropionic acid) [67]. In the postsynaptic plasma membrane, PrP^C^ interacts with co-receptors, LRP1 and metabotropic glutamate receptor 5 (mGluR5). AβOs induce the abnormal accumulation and overstabilization of mGluR5 at the synapse, leading to elevated intracellular calcium levels and increased synaptic toxicity (Figure 5). Notably, AβOs decrease NMDA receptor (NMDAR) intensity at synapses in an mGluR5-mediated manner [68], and they also directly activate NMDAR [69]. Furthermore, the AβO-mediated accumulation of striatal-enriched protein tyrosine phosphatase 61 (STEP61) induces the dephosphorylation of the p-Y14782 of NR2B (a subunit of NMDAR), resulting in an increased internalization of NR1/NR2B (NMDAR subunits) that contributes to the cognitive deficits in AD [70,71].

#### 4.1.3. Nicotinic Acetylcholine Receptor (nAchR)

Acetylcholine receptors (AchR) consist of ionotropic nicotinic AchR (nAchR) and metabotropic muscarinic AchR (mAchR). Among them, soluble Aβ interacts with α7nAchR, which is widespread, has a high relative permeability to calcium, and regulates numerous cellular events in the nervous system [72]. Aβ42 (100 nM), even in the absence of nicotine, has been found to trigger sustained increases in presynaptic calcium levels in the hippocampal synaptosome [73]. Notably, presynaptic responses to Aβ in hippocampal terminals depend on β2nAchR, while those in cortical terminals depend on α7nAchR. These results indicate that specific nicotinic Ach receptors are involved in presynaptic Ca^2+^ regulation in response to Aβ [74] (Figure 5). Conversely, Aβ was observed to inhibit α7nAchR activity at the presynaptic location in rat hippocampal neurons, which evokes a current [75]. Additionally, Aβ42 inhibits nicotinic current by directly blocking the postsynaptic nAchR channels at concentrations as low as 100 nM [76]. Moreover, Aβ42 inhibits Ach release from rat hippocampal synaptosomes, which is activated by K^+^-stimulation. Furthermore, Aβ suppresses nicotine-induced Ca^2+^ accumulation in rat cortical synaptosomes [77]. These controversial results regarding the stimulatory and inhibitory effects of Aβ are concentration-dependent [72]. Notably, an α7AchR partial agonist can release Aβ from α7AchR and enable the partial recovery of α7AchR and NMDA channel function [78].

### 4.2. Aβ Receptors in Endothelia

#### 4.2.1. Low-Density Lipoprotein Receptor-Related Protein 1 (LRP1)

An intracerebral microinjection of Aβ40 in young mice is rapidly cleared from the brain by vascular transport across the blood–brain barrier (BBB) via transcytosis. An antibody targeting low-density lipoprotein receptor-related protein 1 (LRP1) significantly inhibits Aβ clearance. LRP1 is abundant in brain microvessels in young mice but is downregulated in old mice, leading to Aβ accumulation in the brains of AD models [79]. Consistent with these findings, soluble circulating LRP reduces brain levels of Aβ. Furthermore, Aβ can oxidize the LRP1, and the oxidation of LRP1 impairs Aβ clearance [52,80].

#### 4.2.2. The Receptor for Advanced Glycation End Products (RAGE)

RAGE, expressed on endothelial cells, facilitates the influx of circulating Aβ from the blood into the brain [81]. Thus, RAGE may function antagonistically to LRP1. In neurons, RAGE also internalizes Aβ, promoting its intracellular aggregation and accumulation, which rapidly activates p38MAPK and leads to mitochondrial dysfunction [82]. ApoE4 further contributes to the accumulation of Aβ in the brain by binding to and stabilizing AβO, thereby impeding their transition to fibrils. Moreover, apoE4 slows the transport of Aβ across the BBB [83].

## 5. Neuroinflammation

Neuroinflammation has long been associated with AD pathology. Various Aβ complexes interact with microglial and astrocytic receptors, promoting the secretion of proinflammatory cytokines and chemokines and generating ROS. In excess, these processes contribute to neurodegeneration [84], with Aβ stimulating microglial astrocytic cells through several receptors. Microglia can clear accumulated Aβ through phagocytosis and initiate inflammation via multiple receptors, including scavenger receptors (SR-AI/II); the scavenger receptor class B type 1 (SCARB-1); CD36; the CD36/CD47/integrin-α6β1 complex; the receptor for advanced glycation end products (RAGE); Fc receptors; formyl peptide receptor 2 (FPR2); toll-like receptors (TLRs) such as TLR2, TLR4, and coreceptor CD14; complement receptors (CRs); and macrophage receptors with collagenous structures (MARCOs) [85,86]. Notably, the triggering receptor expressed on myeloid cells 2 (TREM2), expressed in microglia, is associated with an increased risk of developing late-onset AD [87].

### 5.1. Aβ Receptors in Microglia and Astrocytes

#### 5.1.1. Complement Receptor CR3

**Figure 6:**
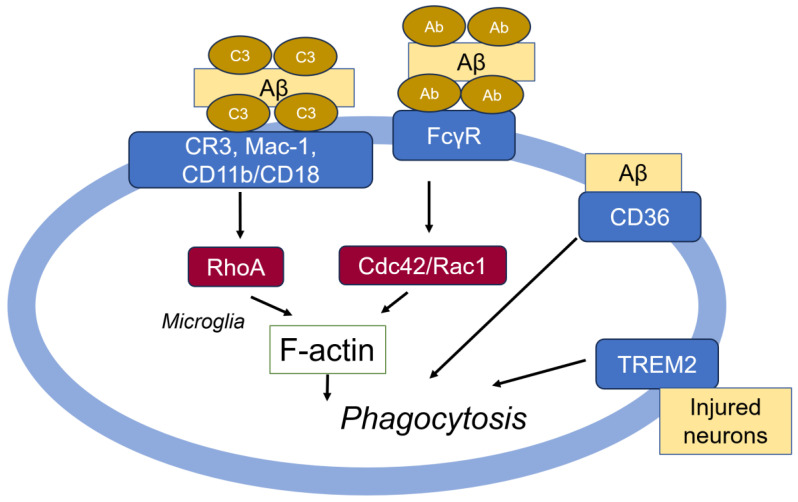
Aβ-induced ROS production and NF-kB activation via multiple receptors in microglia and astrocytes. In the early phases of AD, Aβ activates microglia and astrocytes, which attempt to eliminate Aβ deposits through various receptors via the process of phagocytosis. The complement receptor 3 (CR3, also known as Mac-1, integrin αM/integrin β2, and CD11b/CD18) binds to C3-opsonized Aβ aggregates to facilitate their phagocytosis. Similarly, FcγR binds to antibody-opsonized Aβ aggregates, CD36 interacts directly with Aβ aggregates, and TREM2 binds to debris from injured neurons. Together, these receptors contribute to the clearance of Aβ and neuronal debris.

The complement factor C3 is essential for inducing the phagocytosis of pathogens through interaction with the CR3 receptor, also known as CD11b/CD18, Mac-1, and integrin αM/integrin β2 [85]. In AD patients, the expression of CR3 in microglia is upregulated in the brain. CR3 also binds to factor H, a complement control soluble glycoprotein (155 kDa) circulating in human plasma, heparan sulfate proteoglycans (HSPGs), and Aβ plaques [88]. Furthermore, C3 deficiency accelerates Aβ plaque deposition and neurodegeneration in APP transgenic mice [89]. Antibodies targeting CR3 (CD11b and CD18) reduce Aβ uptake in the microglia [90]. Similarly, the knockdown or deficiency of C3, CR3, and SR-A diminishes Aβ uptake in the microglia, with injected Aβ being higher in C3 and CR3 knockout mice compared to wild-type (WT) mice [91]. These findings demonstrate that C3 and CR3 are crucially involved in Aβ phagocytosis and clearance by the microglia. CR3-mediated phagocytosis is regulated through RhoA GTPase, which controls the dynamics of actin filaments [92] (Figure 6).

#### 5.1.2. FcRs (Fc Receptors)

FcRs in microglia mediate Aβ phagocytosis in the presence of antibodies [93]. Additionally, intracranially administered anti-Aβ antibodies can reduce Aβ deposition either independently or dependently of microglial activation [94]. Notably, lecanemab, a humanized IgG1 monoclonal antibody that binds with high affinity to Aβ-soluble protofibrils, has shown to slow cognitive and functional decline in AD patients [95]. As a result, lecanemab is being used as an anti-AD drug in early-AD patients [96]. FcR-mediated phagocytosis is regulated through Cdc42 and Rac1 GTPases, which control the dynamics of actin filaments [92] (Figure 6).

#### 5.1.3. Formyl Peptide Receptors (FPRs)

Formyl peptide receptors (FPRs), members of the G-protein coupled receptor family, interact with Aβ. The Aβ and FPRL1 complex in humans, a counterpart of FPR2 in mice, is internalized into the cytoplasmic compartment of macrophages and HEK293 cells overexpressing FPRL1. Additionally, on mononuclear phagocytes, Aβ induces IL-1β and superoxide secretion. Notably, the natural Aβ variants Aβ11-40 and Aβ17-40 are potent activators of mouse and human FPR1 at nanomolar concentrations, contributing to neuroinflammation in AD via interaction with Aβ [97]. It is well-established that FPRs can activate several receptor-dependent signal transduction pathways essential to neuroinflammation and neurodegeneration. Accordingly, the pharmacological inhibition of FPRs has been proposed to suppress neuroinflammation and may present a therapeutic strategy [98].

#### 5.1.4. Scavenger Receptor A1 (SCARA1), SCARA2, and CD36

Scavenger receptor A1 (SCARA1) and SCARA2 have a high affinity for both soluble and fibrillar Aβ, facilitating its phagocytosis and clearance from the brain [99]. CD36, also known as SCARB3, fatty acids translocator (FAT), PAS4, and SR-B2, is a type B scavenger receptor that is widely distributed in the human body [100]. It is particularly abundant in blood-borne monocytes/macrophage in the brain under pathological conditions [101] and microglia [102]. Although resting astrocytes do not express CD36, Aβ can elevate the expression levels of CD36, CD47 and RAGE, which mediate Aβ phagocytosis [103] (Figure 6). Notably, CD36 binds and internalizes various ligands, including long-chain fatty acids, advanced glycation end products, oxidized low-density lipoproteins, hydrophobic peptides, apoptotic cell fragments, and antigens from bacteria and parasites [104]. Additionally, CD36 serves as a receptor for thrombospondin and other matrix molecules, playing a role in apoptotic signaling in endothelial cells [105] and macrophages [106]. Remarkably, cholesterol, a major component in lipid metabolism, influences the APP cleavage by β- and γ-secretase, leading to the generation of Aβ fragments. In the early phases of AD, microglia activated by Aβ attempt to eliminate Aβ deposits by inducing CD36 expression and stimulating phagocytosis in astrocytes and microglia [103,107,108]. Aβ phagocytosis due to increased CD36 expression is mediated by PPARγ/RXRα [109]. Concurrently, CD36 binding to Aβ fibrils activates the microglia, putting them in a proinflammatory state, resulting in the production of oxidants [110] and the secretion of ROS and proinflammatory factors such as IL1β and TNFα [111], as well as chemokines like CCL-3, CCL-4, and CXCL-1 [112]. Notably, NRF2, activated by the oxidation of its binding protein KEAP1 [113], increases CD36 expression upon the phagocytosis of myelin debris in macrophages and microglia [114]. These findings suggest that CD36 is crucial for clearing myelin debris and suppressing neuroinflammation. Moreover, CD36 expression in the microglia is strongly correlated with AD pathological features in various brain regions across different mouse models, including TgAPP/PS1 and TgAPP/PS1dE9 [112].

#### 5.1.5. Triggering Receptor Expressed on Myeloid Cells 2 (TREM2)

Based on genome-wide association studies (GWASs), TREM2 has been proposed to play a critical role in the pathogenesis of AD [115]. TREM2 contributes to susceptibility to late-onset AD [116]. It is mainly expressed in myeloid cells, tissue-specific macrophages [117], and microglia in the brain [116]. TREM2 is associated with phagocytosis (Figure 6); anti-TREM2 monoclonal antibodies reduce the engulfment of apoptotic neurons in microglia [118]. Similarly, TREM2 knockdown in microglia decreases both microglial activation and the phagocytosis of injured neurons [119]. Interestingly, the intravenous application of TREM2-transduced myeloid precursor cells facilitates repair within the murine central nervous system by clearing cellular debris during autoimmune encephalomyelitis [120]. TREM2 in the microglia directly binds to AβO with nanomolar affinity, activating TEM2 signaling, while mutations of TREM2 reduce Aβ binding [121]. Aβ enhances TREM2 interaction with its signaling adaptor, DAP12, which regulates the downstream phosphorylation of Syk and GSK-3β. Notably, Aβ increases levels of p-Y525/526 Syk, while TREM2 knockout cells exhibit reduced levels of this phosphorylation. Additionally, shTREM2 increases p-Ser9 GSK-3β (inactive form) in the presence of Aβ in the BV2 microglial cell line, suggesting that TREM2 may activate SYK and GSK-3β [121]. The TREM2-DAP12-DAP10 complex induces a signaling cascade that leads to calcium mobilization, as well as mTOR and MAPK signaling, and the activation of energy metabolism [115]. Notably, the genes with the highest connectivity to TREM2 indicate that it plays a key role in mediating changes in the microglial cytoskeleton necessary for both phagocytosis and migration [116].

### 5.2. Superoxide Generation and Toxicity

**Figure 7:**
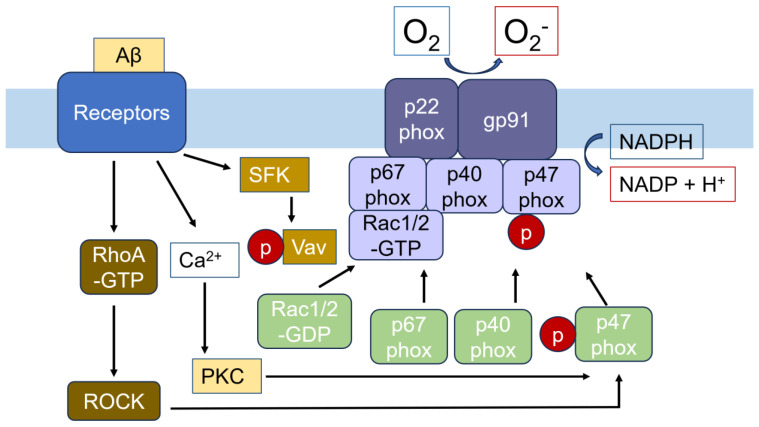
Superoxide generation by NADPH oxidase in microglia and astrocytes in response to Aβ. NADPH oxidase 2 (NOX2) is activated to generate superoxide in response to Aβ. During this process, both the RhoA/ROCK pathway and Ca^2+^/PKC signaling phosphorylate p47phox, a cytosolic component that activates NOX2. Additionally, SFK phosphorylates and activates Vav GEF, which in turn activates Rac, another key activator of NOX2.

It has been well-established that Aβ induces superoxide and neuronal toxicity. NADPH oxidase inhibition by diphynyleneiodonium (DPI) suppresses Aβ-mediated superoxide, while catalase and SOD can mitigate Aβ-mediated neurotoxicity [122]. In microglia, superoxide is primarily generated by NADPH oxidase 2 (NOX2), which is robustly and constitutively expressed, whereas neuronal expression does not appear to be constitutive [123]. Aβ prompts microglia to phagocytose neurons through the activation of NADPH oxidase, likely due to an increase in superoxide intended to impair neurons [124]. Aβ binds to microglial receptors such as CD36, α6β1 integrin, CD47, and class A scavenger receptor (SRA), triggering the activation of Src-family kinases Lyn, Fyn, and Syk. These kinases phosphorylate and activate Vav, a guanine nucleotide exchange factor (GEF) for Rac. This activation leads to subsequent superoxide generation through NADPH oxidase, which consists of membrane components, including gp91 and p22phox, and cytosolic components, such as p40phox, p47phox, and p67phox. Rac1/2 activated by Vav is a critical stimulator for the NADPH oxidase [125]. Notably, Aβ activates RhoA, as well as Rac1 and Cdc42, in BV2 microglial cell lines. RhoA-GTP/ROCK is involved in the phosphorylation and activation of p47phox [126]. Moreover, superoxide induces the phosphorylation of RhoA at the Tyr42 residue, activating ROCK to phosphorylate Ser345 and activate p47phox, thereby upregulating superoxide production and creating a positive feedback loop for superoxide generation [127] (Figure 7).

### 5.3. NF-κB Activation and Production of Proinflammatory Cytokine and Chemokines

In neuroinflammation, NF-κB plays a critical role in coordinating the expression of genes that contribute to both protective and detrimental aspects of the inflammatory response in the nervous system [128]. The binding of nerve growth factor (NGF) to its receptors, such as TrkA and p75NTR, triggers the activation of NF-κB activation, which regulates gene expression [129]. Generally, the brains of individuals with AD exhibit an increased expression and/or activation of NF-κB [130]. Specifically, levels of p65 are found to be elevated in the hippocampus, entorhinal cortex, and frontal cortex of AD patients compared to controls [131,132]. During neuronal loss and instability promoted by Aβ and NFT, NF-κB is central to the vicious cycle of neurodegeneration in AD [133]. NF-κB can regulate Aβ levels, with studies indicating that p65/p50 mediates increases in Aβ in the NT2N human neuronal cell line [134]. Furthermore, the p65 of NF-κB binds to the promoter of BACE1, inducing the expression of β-secretase, which facilitates the amyloidogenic-processing APP [132]. NF-κB also binds to the 5′-regulatory site of the APP promoter, stimulating APP expression [135].

**Figure 8:**
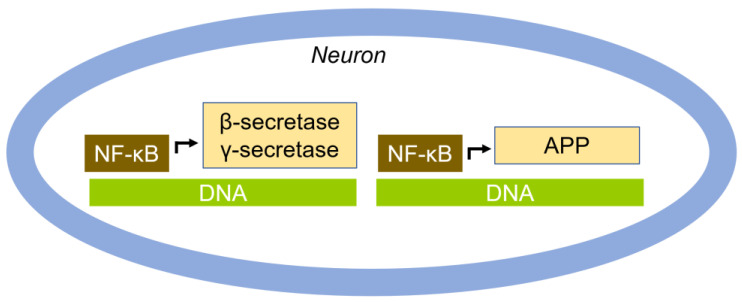
NF-κB-mediated increases in Aβ levels. NF-κB increases Aβ levels by promoting the expression of APP, β-secretase, and γ-secretase through binding to their promoters. The increased β-secretase and γ-secretase activity cleaves APP, resulting in the production of Aβ peptides.

It is noteworthy that NF-κB has dual functions; under the physiological condition, it exerts a repressive effect on Aβ production, lowering the transcriptional activity of the promoters of APP, β-secretase, Aph-1, Pen2, nicastrin, presenilin-1 or 2, and the γ-secretase. However, in Swedish APP-expressing HEK293 cells, NF-κB activates the transcription of APP, BACE1, and γ-secretase, resulting in elevated levels of Aβ [136] (Figure 8).

**Figure 9:**
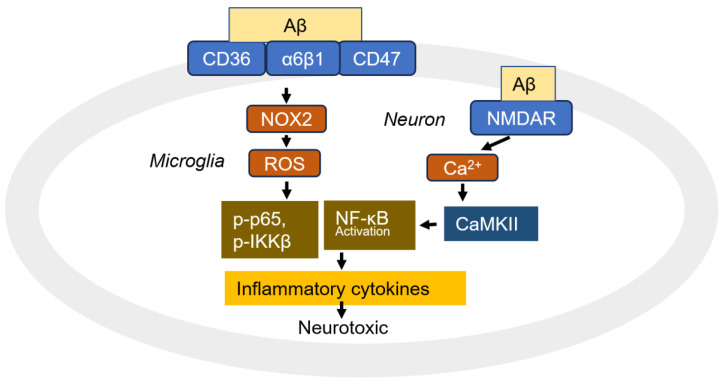
Aβ-mediated activation of NF-κB. Aβ induces superoxide generation, as shown in Figure 7. ROS and Ca^2+^/CaMKII subsequently activate NF-κB, leading to the production of neurotoxic inflammatory cytokines. The detailed mechanism of ROS-mediated NF-κB activation is as follows. Phosphorylation at Tyr42 of IκB and Ser/Thr residues within the C-terminal PEST (Pro-Glu-Ser-Thr) domain of IκB plays a critical role in NF-κB activation. Additionally, hydrogen peroxide increases p-Tyr levels by inhibiting tyrosine phosphatase, thereby promoting NF-κB binding to specific gene promoters. In a separative pathway, hydrogen peroxide directly activates IKK, leading to increased phosphorylation and the subsequent degradation of IκB. An alternative IκB-independent pathway involves hydrogen peroxide-induced phosphorylation of p105, a precursor protein p50, facilitating their cleavage into active NF-κB subunit. Additionally, hydrogen peroxide may stimulate p65 phosphorylation, further contributing to NF-κB activation.

In turn, Aβ activates NF-κB in neurons, glial cells, and the brains of individuals with AD. However, the activation of NF-κB is dependent on factors such as Aβ dosage, brain cell types, and age [130]. Low doses of Aβ (0.1–1 μM) activate NF-κB, increasing immunoreactivity and protecting neurons and granule cells from the toxic effect of higher concentrations of Aβ (10 μM) [137,138]. In rat cortical embryonic neurons, high concentrations of Aβ (25 or 50 μM) reduce levels of constitutive p-p65-mediated NF-κB activation due to the upregulation of IκB levels, which retain cytoplasmic NF-κB [139]. Meanwhile, APP has been shown to positively regulate NF-κB; diminished APP levels lead to a significant decrease in NF-κB activation, whereas APP overexpression markedly increases NF-κB activity [136]. Aβ40 activates the NF-κB pathway through selectively inducing the nuclear translocation of the p65 and p50 subunits, promoting an apoptotic gene expression profile, including an increase in cytochrome C released from the mitochondria in rat primary neuron and human NT2A neuronal cells [134]. Interestingly, the p65/p50 dimer contributes to the pro-apoptotic pathway, while the dimer containing c-Rel plays a neuroprotective role [140,141]. In neurons, NF-κB activation appears to be mediated by both Ca^2+^ and ROS [130]. NF-κB is sensitive to Ca^2+^ signals and is activated in response to elevated intracellular Ca^2+^ levels [142,143]. The gating of NMDA receptors generates the primary glutamate-mediated calcium influx, which is implicated in NF-κB activation [144]. AβOs directly activate NMDA receptors, suggesting that Aβ enhances intracellular Ca^2+^ concentration [69]. During this process, calcium/calmodulin-dependent protein kinase II (CaMKII) is critical for the glutamate-mediated activation of IκB kinase (IKK) and NF-κB [145]. CaMKII is critical for CARMA1-mediated NF-κB activation in T-cell activation [146]. CaMKII also binds to IRAK1, a scaffold protein, and directly phosphorylates p65/RelA at Ser536, thereby activating NF-κB [147]. CaMKIIδ directly interacts with and phosphorylates IKKβ, leading to IκB degradation in cardiac fibroblasts [148]. Additionally, CaMKIV interacts directly with NF-κB and phosphorylates Ser535 in p65, enhancing target gene expression [149] (Figure 9). It is well-established that ROS is an initiator for NF-κB transcriptional regulation [47]. NF-κB activation by Aβ may be mediated through mitochondrial ROS [150]. However, in microglia, which are macrophage-like cells residing in the brain, ROS are primarily generated by NADPH oxidase 2 (NOX2) [123]. Fibrillar Aβ (fAβ) recognizes a complex of receptors, including CD36, α6β1 integrin, and CD47, in the microglia (Figure 9). This complex triggers ROS production, cytokine expression, and the induction of phagocytosis through a tyrosine kinase signaling cascade [151]. Additionally, integrins αLβ2 and αMβ2 are also proposed to be fAβ-binding proteins in the microglia [152].

## 6. Tau Phosphorylation

**Figure 10:**
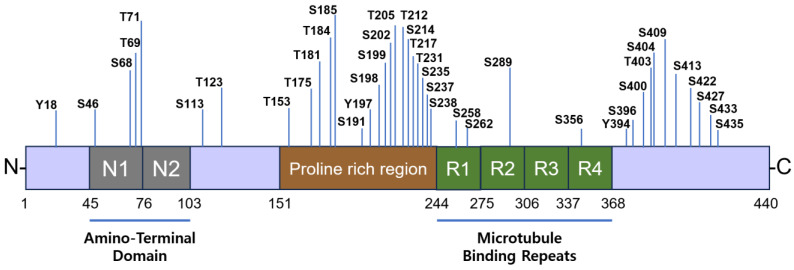
Phosphorylation sites of Tau by various kinases. The Tau protein consists of several domains, including N1, N2, the protein-rich domain, and microtubule-binding repeats (R1-R4). Phosphorylation at residues within the microtubule-binding repeats domain (R1-R4) is believed to cause the dissociation of Tau from microtubules.

Tau phosphorylation is regulated by the balance between the activities of Tau kinases and phosphatase activities. Numerous kinases that phosphorylate Tau at different sites have been identified (Figure 10). Specific phosphorylated sites are found exclusively in AD patients, while some phosphorylated sites are observed in both normal controls and AD patients [153]. Various kinases that phosphorylate Tau protein have been documented, including GSK-3β, CDK5, MAPK (p38, ERK1/2 and JNK), casein kinase (CK), DYRK1A, MARK, PKA, PKB, PKC, CaMKII, and tyrosine kinases such as Src, Fyn, and c-Abl [153,154]. Particularly, Aβ increases phosphorylated Tau at Ser262 and Ser396 residues by GSK-3β and CDK5 [24,25,26]. Furthermore, Aβ modulates phosphorylated Tau through the regulation of protein kinases and protein phosphatases [18].

### 6.1. Glycogen Synthase Kinase-3β (GSK-3β)

**Figure 11:**
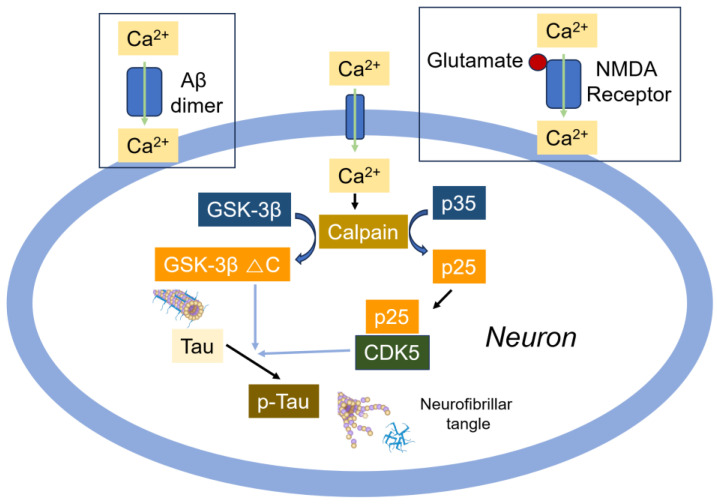
Tau phosphorylation by GSK-3β and CDK5. The uptake of Ca^2+^ activates calpain, a protease that becomes active upon interacting with Ca^2+^. Calpain cleaves GSK-3β and p35, a co-activator of CDK5. As a result, activated GSK-3β and CDK5 phosphorylate Tau, leading to the aggregation of phosphorylated Tau (p-Tau) and the formation of neurofibrillary tangles.

GSK-3β was initially known as Tau protein kinase I (TPK-1) during the early stage of protein purification [155]. The exposure of neurons to Aβ increases GSK-3β activity by inhibiting the PI3-kinase signaling pathway [156]. The initial phosphorylation of Tau by CaMKII is followed by the additional phosphorylation of Tau by GSK-3β at Thr231, Ser235, Ser396, and Ser404 residues depending on the number of repeats, the number of N-terminal inserts, and the initial phosphorylation state [157]. Interestingly, calpain I, a calcium-dependent, non-lysosomal cysteine protease, cleaves GSK-3β at the C-terminus, thereby increasing kinase activity. The truncation of GSK-3β is positively correlated with the overactivation of calpain I and Tau hyperphosphorylation in the AD brain [158] (Figure 11). The interaction between presenilin 1 (PS1) and N-cadherin at the synapse is considered to be neuroprotective by activating the PI3K/Akt survival signaling pathway [159]. By activating PI3K/Akt signaling, PS1 promotes the inactivation GSK-3 through its phosphorylation, which suppresses GSK-3-dependent Tau phosphorylation in AD [159]. In neurons, PS1 also interacts with β-catenin in addition to N-cadherin; this complex is essential for neuronal viability and synaptic plasticity. When GSK-3β phosphorylates PS1 at the Ser353/357 residues, the phosphorylated PS1 reduces its binding to N-cadherin and β-catenin, impairing survival signaling in neurons and thereby contributing to AD [160]. GSK-3β stimulates Aβ production through the upregulation of β-secretase (BACE1) expression, which is regulated by NF-κB [161]. The proposed mechanism involves GSK-3β phosphorylating IKKγ/NEMO at Ser 8, 17, 31, and 43 within its N-terminal domain, which stabilizes IKKγ/NEMO and activates NF-κB [162]. Additionally, GSK-3β activates NF-κB by the phosphorylation of the p65/RelA subunit in response to lipopolysaccharide (LPS) in the microglia, inducing the expression of proinflammatory cytokines and chemokines [163,164]. Notably, GSK-3β is linked to p65 acetylation, which influences p65 transactivation activity [163].

### 6.2. CDK5

In contrast, CDK5 is essential for both embryonic corticogenesis and neurogenesis in the adult hippocampal and dentate gyrus. The CDK5-activating cofactor p35, but not p39, predominantly regulates neurogenesis in the adult subgranular zone (SGZ) [165]. Interestingly, however, it has also been reported that p39, but not p35, significantly enhances CDK5 activity during neuronal differentiation in rat and mouse [166]. The phosphorylation of Tau at Ser396/404 residues in response to Aβ is associated with CDK5 and its activator p35. CDK5 exhibits dual roles, promoting either neuronal survival or death. Neuronal death due to the dysregulation of CDK5 is linked to the calpain-mediated cleavage of its coactivator, p35 or p39, into p25 and p29, respectively [167]. The role of p25/CDK5 has been implicated in neurodegenerative diseases, as it may induce or accelerate the formation of early neurofibrillary tangles, primarily composed of hyperphosphorylated and aggregated Tau [168,169] (Figure 11). However, the CDK5 inhibitor roscovitin abolishes p-Tau Ser396/404, indicating that CDK5 contributes to p-Tau Ser396/404 formation [170]. Nestin functions as a selective scaffold for doublecortin (CDX) with activated CDK5/p35, facilitating CDX phosphorylation by CDK5/p35. An overexpression of nestin reduces axonal growth cone size and the number of filipodia. Additionally, in the presence of nestin, Sema3a significantly reduces both axonal growth cone size and the number of filipodia [171].

### 6.3. Microtubule Associated Proteins (MAP)/Microtubule Affinity-Regulating Kinase (MARK)

MARKs phosphorylate Tau at serine residues, including Ser262, Ser293, Ser324, and Ser356, promoting Tau’s release from microtubules and leading to its aggregation [153]. MARK2 is activated through phosphorylation at Thr208 in the activation loop by two kinases: MARKK/TAO-1 [172] and liver kinase B1 (LKB1) [173]. LKB1 also enhances the activities of MARK1, MARK3, and MARK4, as well as MARK2 [173]. However, the phosphorylation of Ser212 in MARK2 inhibits its activity [173]. GSK-3 directly phosphorylates MARK2 at Ser212, thereby inhibiting its kinase activity [174]. Both CDK5 and LKB1 phosphorylate MARK4 in the activation loop and spacer domain, respectively, leading to its activation. Subsequently, both CDK5 and MARK4 induce the phosphorylation of Tau at the Ser262 residue [175]. Interestingly, MARK2 knockout mice exhibit leaning impairment [176], suggesting that MARK2 inhibitors may not be suitable as therapeutic agents for AD. The two peptide sequences in the KXGS motif of Tau—258-SKIG**S**-262 and 352-SKIG**S**-356—are identical. MARK4 phosphorylates both Ser262 and Ser356 within microtubule-binding repeats [177]. Consequently, the monoclonal antibody 12E8 can recognize both p-Ser262 and p-Ser356 on Tau [178]. Phosphorylated Tau at these sites has been known to regulate its microtubule binding, intracellular localization, and protein–protein interactions [179]. Phosphorylated Tau at these sites is found in pre-neurofibrillary tangles and is associated with enhanced seeding potency, suggesting an initiating role in Tau abnormalities [180,181].

### 6.4. Rho-Associated Protein Kinase (ROCK)

Recently, the effects of ROCK inhibitors on p-Tau have also been studied using a human neuroblastoma cell line (M1C) expressing wild-type Tau protein, primary mouse neurons, and a mouse model of Tauopathy (rTg4510 line). ROCK inhibitors inactivate GSK-3β and CDK5 while activating protein phosphatase 2A. Additionally, ROCK inhibitors promote autophagy and proteasome pathways, potentially enhancing Tau degradation and thereby reducing total Tau protein levels [182]. It has been reported that ROCK phosphorylates Tau at Thr245, Thr377, and Ser409 residues [183]. However, it was proposed that ROCK indirectly phosphorylates Tau via the ROCK/Akt/GSK-3β/p-Tau signaling pathway [182].

### 6.5. Dual-Specificity Tyrosine Phosphorylation-Regulated Kinase 1A (DYRK1A)

DYRK1A, which is overexpressed in Down syndrome (DS), may play a significant role in brain developmental defects and contribute to early-onset neurodegeneration, neuronal loss and dementia in DS [184]. In vitro, DYRK1A phosphorylates Tau at the Thr212, Ser202, and Ser404 residues. DYRK1A transgenic mice overexpressing this kinase exhibit phosphorylated Tau protein at the Thr212 residue in the brain. Cells from DYRK1A-transgenic mice also show p-Ser202 and p-Ser4040 Tau [185]. Additionally, overexpressed DYRK1A enhances the phosphorylation of APP at the Thr668 residue [185], facilitating its cleavage by BACE1 [19] and γ-secretase [186], which increases Aβ40 and Aβ42 levels and leads to brain β-amyloidosis [184]. A selective oral inhibitor for DYRK1A, SM07883, has been proposed as a potential therapeutic for AD [187]. Recently, several inhibitors aimed at disrupting the DYRK1A-induced hyperphosphorylation of Tau and APP have been screened using a molecular modeling approach [188].

## 7. Regulation of Neuronal Function by RhoA GTPases

### 7.1. Regulation of RhoA GTPase Activity

**Figure 12:**
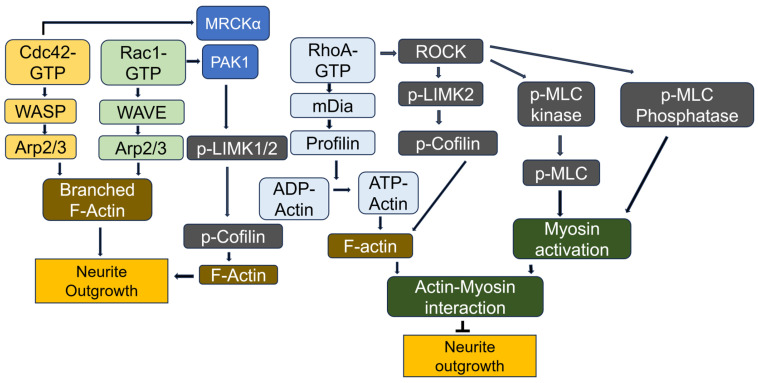
Signaling pathways of RhoA, Cdc42 and Rac1 in regulating cytoskeletal structure. RhoA promotes actin filament formation through the mDia/profilin/ATP-actin and ROCK/LIMK/p-cofilin pathways. At the same time, RhoA enhances myosin activity via the ROCK/MLC kinase and ROCK/p-MLC phosphatase pathway. The interaction between F-actin and myosin consequently leads to cell contraction. In contrast, Cdc42 and Rac promote actin filament formation through the activation of MRCK and the PAK1/LIMK/p-cofilin pathways. Additionally, Cdc42 and Rac1 stimulate the formation of branched F-actin filaments through the activation of WASP and WAVE, facilitating the development of multiple branched actin filaments and promoting cell protrusion.

Once activated, Rho GTPases interact with effector proteins that propagate signals to downstream targets, facilitating various cellular responses. Key effectors include two Rho-associated coiled-coil kinases: ROCK1 and ROCK2. The Rho-binding domain (RBD) in the carboxyl-terminal region of Rho-kinase functions as an autoinhibitory loop that folds back onto the kinase domain, inhibiting the kinase activity of ROCK. GTP-bound RhoA binds to the RBD of ROCK, exposing the catalytic domain and making it accessible to its substrates, thereby activating ROCK [189]. Rho-activated ROCK phosphorylates myosin phosphatase, myosin light chain kinase (MLCK) and LIM-kinase 2 (LIMK2), promoting actin filament formation and enhancing actin–myosin interactions via cofilin phosphorylation. Cofilin typically functions to sever F-actin, but when phosphorylated by LIMK, it becomes inactive, leading to stabilized F-actin [190]. In contrast, six p21-activated protein kinases, PAK1-6, are activated by Cdc42 and Rac1. LIMK1 is activated by PAK1, further contributing to cytoskeletal changes [191]. The Rac1/Pak1/LIMK1 signaling pathway regulates cofilin activity within the lamellipodium [192]. Furthermore, PAK1 is essential for axon and dendrite specification, contributing to neuronal polarization and differentiation in hippocampal neurons [193]. Additionally, myotonic dystrophy kinase-related Cdc42-binding kinase α (MRCKα), an effector protein kinase of Cdc42, phosphorylates LIMK2 at Thr505 within the activating segment, promoting cofilin phosphorylation and cytoskeletal reorganization [194]. PI(4,5)P2 is involved in actin nucleation through the activation of the Cdc42, N-WASP (neuronal Wiskott–Aldrich syndrome protein), and Arp2/3 (actin-related protein2/3) complex [195,196]. Additionally, WAVE (WASP family verprolin-homologous protein), activated by Rac, induces actin filament clusters, and profilin is essential for this actin polymerization [197]. Furthermore, Rac1 activates the WAVE regulatory complex (WRC), driving Arp2/3 complex-mediated actin polymerization that underpins various cellular processes [198]. Notably, Rac- and Arp2/3-mediated actin networks may directly antagonize Rho signaling [199]. The Arp2/3 complex acts as an actin nucleator, organizing filaments into branched networks [200] (Figure 12).

### 7.2. RhoA Effects on Neurite Outgrowth in Neuronal Cells

There is compelling and increasing evidence that Rho GTPase family proteins and their related molecules play critical roles in various aspects of neuronal development, including neurite outgrowth and differentiation, axon pathfinding, and the formation and maintenance of the dendritic spine [201]. Specifically, RhoA has been widely regarded as a key molecular switch that inhibits neurite outgrowth, while Cdc42 and Rac serve as positive regulators of neurite outgrowth and dendritic spine formation [202]. The RhoA effects on neurite outgrowth have been investigated in several types of neuronal cells. For instance, serum-starved mouse N1E-115 neuroblastoma cells undergo neurite formation, which depends on the activation of Rac and Cdc42 [203,204]. In PC12 cells, a dominant-negative RhoA mutant (N19), but not a constitutively active form (V14), stimulates the initiation, elongation and branching of neurite growth in response to NGF [205]. Consistent with these findings, the inhibition of Rho using C3 toxin in PC12 cells and primary retinal neurons promotes neurite growth on inhibitory substrates [206]. Additionally, the microinjection of the catalytic domain of ROKα (ROCKII) and RhoA V14 rapidly induces neurite retraction in PC12 cells upon NGF stimulation [207]. Lysophosphatidic acid (LPA)-induced neurite retraction is well-documented. The mechanism involves LPA and Gα12/Gα13 activating endogenous RhoA, which inhibits neurite outgrowth and induces neurite retraction and growth cone collapse in N1E-115 cells [208]. ROCK activates myosin II by phosphorylating Ser19, which initiates contraction between and actin filaments in the filopodia within the growth cone. This contraction pulls the actin filaments towards the center of growth cone and away from the direction of protrusion, ultimately leading to growth cone collapse [209,210]. Additionally, ROCK phosphorylates the myosin-binding catalytic subunit of myosin phosphatase, inactivating it and leading to myosin activation through an increase in the phosphorylated myosin level [211]. Although many studies have shown that active RhoA inhibits axonal growth, the precise molecular mechanism remains unclear. Recently, Bradke and colleges reported that RhoA-mediated myosin II activation interacts with actin filament to form arc-like barriers, which obstruct microtubule advances within the neuronal growth cone [212]. They further observed that RhoA functions oppositely in neurons and astrocytes: neuronal RhoA suppresses axonal regeneration, whereas astrocytic RhoA decreases inhibitory astrocyte reactivity. Accordingly, they proposed that only neuron-specific RhoA should be inhibited to promote axonal regeneration [213].

### 7.3. Investigation of RhoA Functions in Neurons Using Animal Model

Studies on RhoA function in neurons have also been also investigated using animal models. Knock-in mice with dominant negative RhoA (N19RhoA) show significant increases in the density and absolute number of neurons in the somatosensory cortex compared to wild-type littermates, potentially due to decreased neuronal apoptosis during early postnatal development [214]. After peripheral nerve injury, RhoA knockout in the motor neurons reduces dendritic degeneration, while promoting its regeneration [215]. In general, RhoA is established as a negative regulator of neuriotogenesis, axon formation, and axonal number and length [216]. However, some inconsistencies have been reported, likely due to the versatile functions of RhoA, which supports various neurogenesis steps across different brain cell types in mammal brains. Additionally, RhoA deletion has been shown not to affect the axonal and dendritic projections or distribution of motor neurons [217]. In *Drosophila*, neurons lacking RhoA exhibit dendritic overextension, while activated RhoA expression reduces dendritic complexity, suggesting that RhoA regulates dendritic, but not axonal, morphogenesis [218]. Interaction between ephrin ligands and Eph receptors on presynaptic and postsynaptic membrane surfaces, respectively, regulates synapse formation, function and plasticity in neurons [219]. Notably, RhoA deletion impairs ephrineB3 expression [217], suggesting RhoA’s involvement in synapse formation regulation. During brain development, RhoA loss in neurons has a marginal effect on neuronal migration; however, RhoA deletion in radial glial cells has a significant effect on neuronal migration [220]. Studies using a RhoA deletion mutant reveal that RhoA is essential for ventricular zone organization and for the proliferation, survival and localization of mitotic cells. In RhoA-deficient mice, N-cadherin and β-catenin are reduced in the spinal cord, highlighting RhoA’s role in cell–cell interaction within adherens junctions (AJs) [221]. Consistent with these findings, RhoA accumulates at AJs and colocalizes with the cadherin–catenin complex, an AJ component [222]. Interestingly, mDia, but not ROCK1, is reduced on the ventricular surface of the spinal cord in RhoA-deficient mice [221]. Conditional RhoA deletion in midbrain and forebrain neural progenitors impairs apical AJs and leads to severe brain dysplasia; notably, RhoA-deleted neural progenitor cells show enhanced proliferation [222]. However, RhoA is controversially required for neuroblast proliferation but not for neuronal survival in *Drosophila* [218]. Additionally, RhoA knockout in macrophages has been investigated for its effect on nerve regeneration and Wallerian degeneration—an active process of anterograde degeneration of the distal axon following nerve injury. RhoA deletion in macrophages detrimentally impacts Wallerian degeneration and nerve regeneration, likely due to inhibited macrophage migration and phagocytosis [223]. During neuroinflammation, RhoA activity is downregulated in the microglia; however, neuroinflammation with LPS promotes cell death in RhoA-knockout in the microglia, indicating that a minimal level of RhoA activity is necessary for microglial reactivity and survival during neuroinflammation [224]. Therefore, the development of RhoA-targeted drugs for peripheral nerve injury should account for cellular specificity [223].

### 7.4. Molecular Mechanism of RhoA Inactivation During Neurite Outgrowth

**Figure 13:**
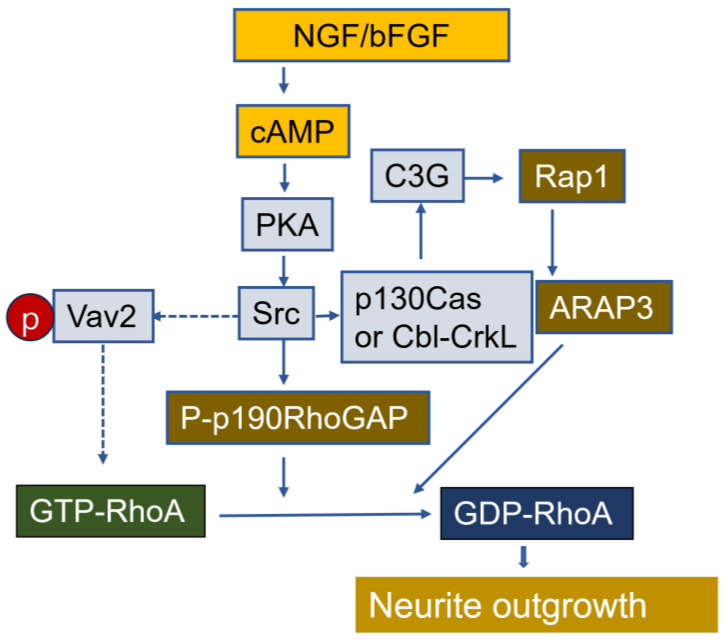
Mechanisms of RhoA inactivation by neurotrophic stimulating factors. During neurite outgrowth in response to NGF, bFGF, and cAMP, RhoA is inactivated through the two main pathways: the Src/p190RhoGAP/RhoA-GDP pathway and the Rap-GTP/ARAP3 (Rap-dependent RhoGAP)/RhoA-GDP.

The NGF-mediated activation of the TrkA receptor promotes both Rac1 activation and RhoA inactivation during the initial phase of neurite outgrowth [225]. Mechanistic studies of neurite outgrowth have shown that NGF, bFGF and cAMP induce neurite extension from PC12 cells by RhoA inactivating RhoA through p190RhoGAP and Rap-dependent RhoGAP (ARAP3) [226,227,228]. Additionally, cAMP induces RhoA phosphorylation at Ser188, promoting its binding to RhoGDI and thereby leading to RhoA inactivation [228] (Figure 13). In the adult nervous system, Rap1 and Rap2 regulate the maturation and plasticity of dendritic spine and synapses [229]. During the multipolar-to-bipolar (MTB) transition, one of the processes becomes axonal, with active Rap1B localizing to the future axon in unpolarized neurons [230]. C3G/RapGEF1 is essential in multipolar neurons for the MTB transition [231]. The activation of EphA4 by ephrins inactivates Rap1 and growth cone collapse in hippocampal neurons through Sapr1, a GTPase-activating protein spine-associated RapGAP that directly interacts with the EphA4 receptor [232]. Dimeric plexins also exhibit GAP activity for Rap1 and Rap2 in response to semaphorin, leading to neuronal growth collapse [233,234]. A deficiency in Epac1/2, Rap GTPase GEFs that are directly activated by cAMP, reduces the number of primary dendrites, dendritic spine density in the hippocampus, and synaptic plasticity [235].

### 7.5. Molecular Mechanism of RhoA Activation in Neurons

The transfection of p75^NTR^ in neuronal cells activates RhoA, whereas neurotrophin binding abolishes RhoA activation [236]. The molecular mechanism of RhoA activation by p75^NTR^ involves p75^NTR^ interacting with the RhoA-RhoGDI complex, acting as a GDI displacement factor (GDF). This interaction facilitates the release of RhoA from the RhoA-RhoGDI complex, leading to RhoA activation. Myelin components, including Nogo, myelin-associated glycoprotein (MAG) and myelin oligodendrocyte glycoprotein (OMgP), reinforce this interaction, preventing neurite outgrowth through RhoA activation [237].

**Figure 14:**
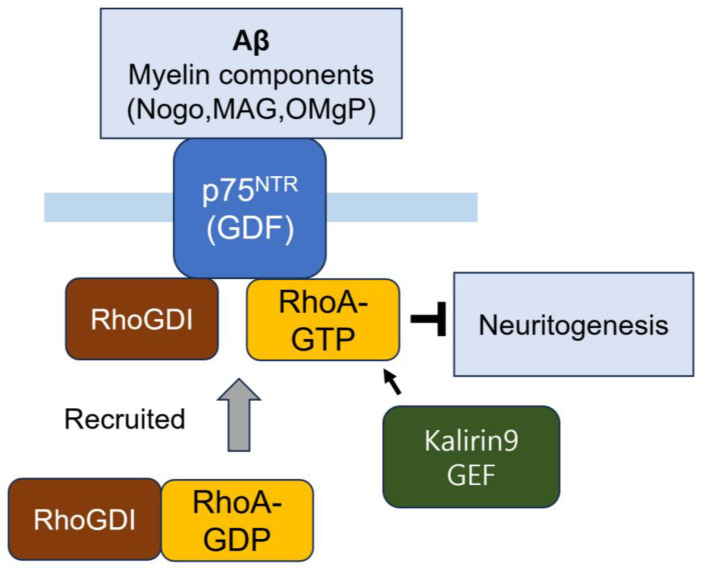
Mechanism of RhoA activation by Aβ and myelin components. Aβ and myelin components, including Nogo, MAG, and OmgP, bind to p75^NTR^ receptor, which has GDF activity. The interaction with the p75^NTR^ receptor causes the dissociation of the RhoA-RhoGDI complex. Subsequently, RhoA GEF Kalirin 9 facilitates the incorporation of GTP into RhoA after the GDP release, impairing neurite outgrowth.

The treatment of the human Aβ42 peptide in p75^NTR^-deficient embryonic mouse hippocampal neurons does not induce significant cell death. Additionally, an injection of Aβ42 into the hippocampus of adult mice leads to the significant degeneration of wild-type, but not p75 ^NTR^-deficient, cholinergic basal forebrain neurons, indicating that the p75 ^NTR^ is a key mediator of Aβ-induced toxicity. [238]. In PC12 nnr5 cells, which lack a TrkA receptor, Aβ greatly increases RhoA activity, but NGF administration prevents the activation of RhoA in response to Aβ [239]. This suggests that Aβ competes with NGF for binding to p75^NTR^, but not to TrkA [240,241]. Aβ binding to p75^NTR^ in rat cortical neurons specifically induces neuronal death [240]. Specifically, Aβ binding to trimeric or monomeric p75^NTR^ in neurons strongly induces the transcription of c-Jun and the stimulation of JNK [241]. Aβ activates the RhoA by binding to p75^NTR^, which leads to the suppression of the NGF-induced activation of protein phosphatase 1B (PTP1B), which is required for neuronal survival. Kalirin9, a dual RhoGEF, directly binds to p75^NTR^, stimulating p75^NTR^-Nogo receptor-dependent RhoA activation, which inhibits neuronal outgrowth in response to MAG [242] (Figure 14). The expression of p75^NTR^ is notably increased in AD hippocampal neurons, with this increase attributed to proNGF accumulation and ROCK activation [243]. Interestingly, the pharmacological inhibition of RhoA with C3-ADP ribosyl transferase or the transfection of dominant-negative RhoA or PTP1B in cultured hippocampal neuron protect these neurons from the detrimental effect of Aβ [239]. This finding highlights a potential pathway for AD drug development through RhoA inactivation.

### 7.6. Axon Guidance Molecules Regulate RhoA Activity

Recently, four classes of axon guidance molecules—netrins, slits, ephrins, and semaphorins (Semas) have been investigated [244].

#### 7.6.1. Sema3A

Also referred to as collapsin, Sema3A induces the collapse of neuronal growth cones in embryonic chick brains [245,246]. Receptors for semaphorins are primarily plexin proteins, which often function alongside various co-receptors [247]. Plexins contain a Rho-binding domain (RBD) that interacts with Rho family GTPases [248]. It has been suggested that the plexin A or B family may bind to and sequestrate Rac1 away from PAK, leading to F-acin disassembly [249].

#### 7.6.2. Eph (Erythropoietin-Producing Hepatocellular Carcinoma) Receptors

**Figure 15:**
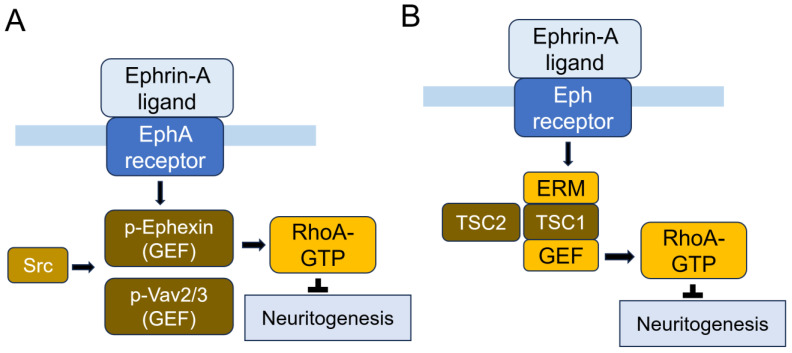
Mechanisms of axon guidance by ephrin-A in inhibiting neuritogenesis. (**A**) Ephrin-A binds to the EphA receptor, activating Src, which in turn phosphorylates and activates ephexin and Vav2/3, leading to RhoA activation. (**B**) In an alternative pathway, ephrin-A binds to the EphA receptor, and TSC1 interacts with a GEF for RhoA, resulting in RhoA activation.

Eph receptors exhibit receptor tyrosine kinase activity and are classified into two classes: A and B types. The binding of Eph receptors to ephrin ligands initiates a signaling cascade in the Eph receptor-carrying cells, referred to as forward signaling. Additionally, ephrin ligands, when binding with Eph receptors, can also act as receptors and transduce signals in ephrin-ligand-containing cells, known as reverse signaling [250]. Several components of the ephrin-A system, including EphA1, EphA4, ephrin-A1 and ephrin-A5, have been associated with neurodegenerative conditions such as AD or amyotrophic lateral sclerosis [250]. Specifically, EphA4 serves as a substrate for γ-secretase, a protease that malfunctions in many early-onset AD cases, while the overexpression of EphA4 increases the number of dendritic spines by activating the Rac signaling pathway [251]. Additionally, the EphA4-Lyn pathway plays a crucial role in APP metabolism, generating the APP C-terminal fragment (C99), the APP intracellular domain (AICD), and the Aβ peptide [252]. Concerning Rho GTPase’s involvement in EphA signaling pathway, activated EphA receptors recruit Src kinases, ephexin families, and Vav2/Vav3. Ephexins are guanin nucleotide exchange factors (GEFs) for RhoA. The overexpression of ephexin induces growth cone collapse, whereas a GEF-inactive ephexin mutant inhibits ephrin1-induced growth cone collapse [253]. The phosphorylation of ephexin at the tyrosine residue enhances its GEF activity toward RhoA, resulting in Eph-mediated growth cone collapse in neurons [254] (Figure 15). The activation of EphA receptors by ephrin-A ligands in neurons leads to a decreased inhibition of TSC2 (tuberous sclerosis complex 2, also known as tuberin), indicating that TSC2-Rheb-mTOR signaling works in concert with the ephrin-Eph receptor system to control axon guidance [255]. Recent research showed that TSC2 activates RhoA, leading axon growth cones to collapse upon ephrin-1 binding [256]. TSC2 is a GTPase-activating protein (GAP) toward Rheb that promotes the phosphorylation of mTOR and p70 ribosomal protein S6 kinase 1 (S6K) and eukaryotic initiation factor 4E binding protein (4EBP) [257]. However, in response to growth-stimulating signals, such as insulin, TSC2 becomes inactivated by Akt-dependent phosphorylation, which destabilizes TSC2 and disrupts its interaction with TSC1, leading to protein synthesis during cell growth [257]. Notably, TSC1, also known as the tumor suppressor hamartin, binds to the ERM proteins and RhoA, activating RhoA and promoting cell adhesion [258]. It is speculated that TSC1 interacts with RhoA GEF [258] (Figure 15).

#### 7.6.3. Netrin-1

Netrin-1 triggers either the attraction or repulsion of axon growth cones through specific intracellular signaling responses [259]. Receptors that bind netrin orchestrate cytoskeletal and cell membrane remodeling [259]. Netrins function by binding to receptors such as DCC (deleted in colorectal cancer) and UNC5, which activate distinct signaling cascades. The binding of netrin-1 to homodimeric DCC/DCC typically promotes cell survival and axon attraction [260]. In the absence of netrin-1, UNC5B (also known as p53RDL1) mediates p53-dependent apoptosis, whereas interaction with netrin-1 inhibits p53-induced apoptosis [261]. The netrin-1/DCC/FAK signaling complex recruits RhoGEFs such as DOCK180 (DOCK1) [262] and Trio [263] to activate Rac1. Additionally, Tiam-1 GEF is recruited to activate both Cdc42 and Rac1 [264]. Conversely, the interaction of netrin-1 with heterodimeric UNC5/DCC or with UNC5 alone can mediate repulsive guidance cues [265] and may induce apoptosis through pathways involving DAPK1 (Death-associated Protein Kinase 1) and caspases, depending on the cellular context [266,267]. Netrin stimulates Src-mediated phosphorylation of UNC5 at Y482 (Y568 in murine UNC5C), which is essential for repulsive axon guidance in vivo [268,269]. Netrin-1 reduces the interaction of UNC5C with polymerized TUBB3, a neuron-specific microtubule subunit, in growth cones, resulting in axon repulsion [270]. Interactions between netrin-1 and UN5/DSCAM (Down syndrome cell-adhesion molecule) also induce a repulsive response [271]. The switch between attraction and repulsion to netrin-1 is regulated by four primary mechanisms: levels of membrane receptors, intracellular secondary messengers (such as calcium, cAMP, and cGMP), netrin-1 concentration, and extracellular environment [259].

#### 7.6.4. Slits

**Figure 16:**
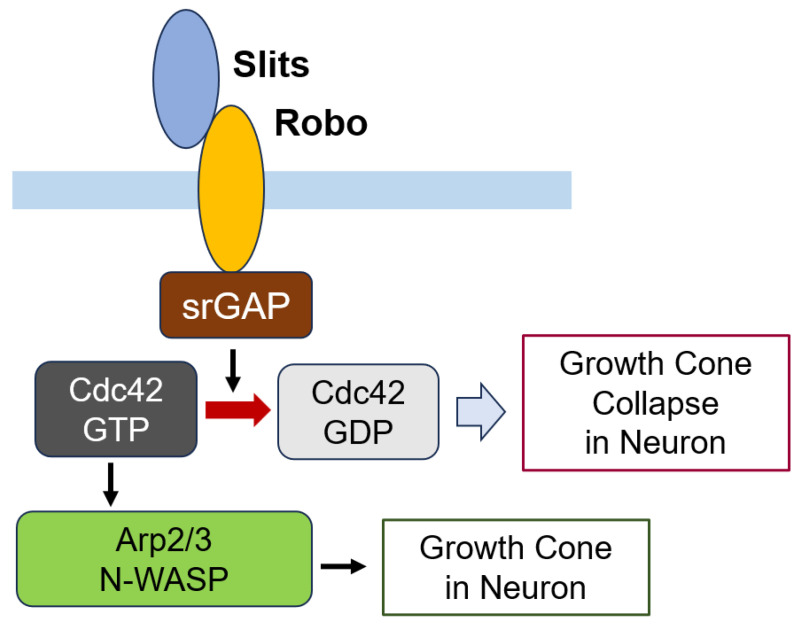
Mechanisms of axon guidance by the slit in inhibiting neuritogenesis. Slit binds to the Robo receptor, recruiting srGAP, which inactivates Cdc42, leading to growth cone collapse in neurons.

Slits are secretory glycoproteins involved in regulating numerous physiological processes. They exert their effects by binding to specific receptors; binding with Robo receptor and plexin A1 regulates axon guidance, while binding with DSCAM1 triggers axon branch extension [272]. Several Slit-Robo GAPs (srGAPs), including srGAP1, srGAP2, srGAP3, and Arhgap4, interact with the intracellular domain of Robo. Upon slit stimulation, srGAP1 interacts with Cdc42 and RhoA, srGAP2 binds to Rac1, and srGAP3 has been reported to interact with both Rac1 and Cdc42 [273]. Consequently, slit binding to Robo results in the inactivation of Cdc42, which suppresses the activation of Arp2/3 and N-WASP, ultimately leading to axon repulsion [274] (Figure 16).

### 7.7. Brain-Derived Neurotrophic Factor (BDNF)

Brain-derived neurotrophic factor (BDNF) is widely expressed in healthy human brains. Recent studies have examined the interactions between BDNF in mature neurons and their roles in synaptic plasticity. BDNF supports neuronal survival and synaptic plasticity, while netrins play roles in axonal guidance and synapse formation. Both are essential for maintaining overall brain health. The pro-BDNF isoform, which contains two sequences (a pro-domain and a mature domain) interacts with specific receptors: sortilin and p75^NTR^ for the pro-domain, and TrkB (tropomyosin receptor kinase B) for the mature domain. The mature domain of BDNF, as the sole component of the m-BDNF isoform, has the highest affinity for TrkB, which undergoes homodimerization and autophosphorylation upon stimulation. The binding of m-BDNF to TrkB promotes anti-apoptotic and pro-survival effects and modulates NMDA receptor-dependent synaptic plasticity [275].

**Figure 17:**
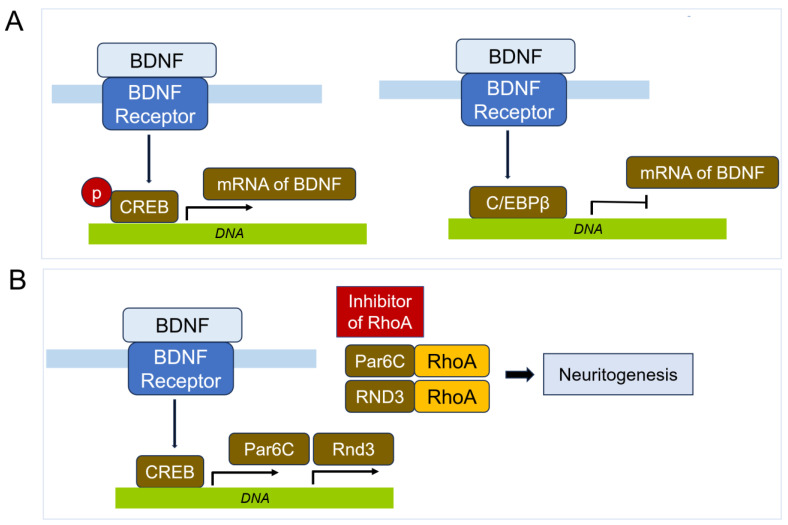
Regulation of BDNF and its role in RhoA inactivation. (**A**) When BDNF stimulates CREB to bind to the promoter of BDNF, BDNF expression is enhanced. In contrast, when the transcription factor C/EBPβ binds to BDNF promoter, it suppresses BDNF expression. (**B**) BDNF binds to its receptor, stimulating transcription factor CREB, which induces the expression of Par6C and Rnd3. These proteins interact with RhoA, leading to its inactivation.

BDNF also plays a significant role in the development of Aβ plaques [276], which are closely associated with neurodegenerative diseases such as AD and Parkinson’s disease (PD). The dysregulation of BDNF can contribute to pathological processes of neuronal damage, promoting the formation of Aβ plaques and subsequent neuronal injury [277,278,279,280,281,282]. BDNF exerts its effect primarily through binding to its high-affinity receptor, TrkB, activating downstream signaling pathways such as the PI3K/Akt pathway, the MAPK/ERK pathway, and the PLCγ pathway [275,283,284,285]. These pathways promote neuronal survival, growth, and differentiation. Specifically, the PI3K/Akt pathway inhibits apoptosis by phosphorylating and inactivating pro-apoptotic proteins. The MAPK/ERK pathway promotes cell survival and synaptic plasticity by modulating gene expression, while the PLCγ pathway leads to the release of intracellular calcium, essential for various cellular processes, including synaptic plasticity. The C/EBPβ (CCAAT/enhancer-binding protein β) transcription factor has been reported to bind to the promoters of both BDNFs, acting as a transcriptional repressor in PD [282]. This binding inhibits the expression of crucial neurotrophic factors, potentially exacerbating neurodegeneration. Understanding the precise mechanisms of this repression and its impact on BDNF pathways could reveal new targets for therapeutic intervention. Research has shown that BDNF not only supports the survival of existing neurons but also encourages the growth and differentiation of new neurons and synapses [286]. Understanding the mechanisms by which BDNF influence neuronal health could provide valuable insights for developing therapeutic strategies for neurodegenerative diseases. Targeting the molecular pathways regulated by BDNF, as well as modulating the activity of transcription factors like C/EBPβ, offers promising approaches to mitigate the progression of neurodegenerative disorder progression. Notably, BDNF is downregulated in the cortex early in AD progression, with AβO reducing cortical BDNF mRNA expression [287]. One potential mechanism is via the downregulation of the phosphorylated cAMP response element-binding (CREB) transcription factor, partly due to interactions between Aβ and PKA [288] (Figure 17). Additionally, levels of TrkB, the BDNF receptor, are decreased in AD, while truncated TrkB.T1, a dominant negative form affecting both TrkB and p75, is elevated [289]. Furthermore, Aβ induces a calpain-mediated cleavage on TrkB receptors, decreasing their availability [290]. In contrast, BDNF exhibits neuroprotective effects against the toxic impact of Aβ peptide [291]. BDNF also prevents the functional integrity of neural networks in β-amyloidopathy models [292]. One suggested mechanism for BDNF’s protective effect is its ability to shift APP processing towards the α-secretase pathway, as observed in neuronal cell lines [293].

BDNF affects RhoA in neurons by producing a rapid and transient increase in RhoA protein levels, along with cofilin phosphorylation and actin polymerization, specifically in the dendritic spines of CA1 and CA3 regions of adult rat hippocampal slices during LTP consolidation [294]. Conversely, the pro-BDNF/p75^NTR^/sortilin complex triggers neuronal apoptosis through activations of JNA, RhoA, and NF-κB [275]. Additionally, the RhoA-JNK signaling pathway is activated when pro-BDNF binds to the p75^NTR^ receptor, contributing to post-stroke depression [295]. The Val66Met polymorphic in the pro-domain region of pro-BDNF induces acute growth cone retraction and reduces hippocampal neuron density [296]. Furthermore, CREB, induced by BDNF, promotes the transcription of RhoA inhibitor proteins, including Par6C (Pard6A) and Rnd3 (RhoE), which are essential for synaptogenesis [297] (Figure 17).

### 7.8. RhoA and Microtubule

**Figure 18:**
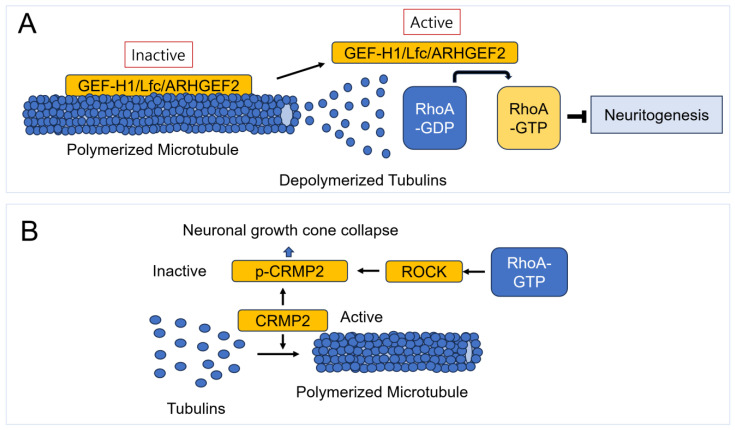
Relationship between microtubules and RhoA. (**A**) In its inactive form, GEH1/Lfc/ARHGEF2 binds to the polymerized microtubules. When microtubules depolymerize, GEF-H1 is released and activated, leading to RhoA activation, which suppresses neuritogenesis. (**B**) CRMP2 facilitates microtubule assembly; however, when phosphorylated by RhoA/ROCK, CRMP2 becomes inactive, promoting microtubule depolymerization.

RhoA activity has been reported to regulate microtubule stability. During the epithelial-mesenchymal transition (EMT) process, RhoA inactivation leads to the destabilization of basal microtubules [298]. In cultured hippocampal neurons, RhoA activity is selectively localized in growth cones of undifferentiated neurites. However, in developing neurons, RhoA activity is low in nascent axons and high in elongating axons. RhoA-ROCK signaling prevents axon initiation, while the actin polymerizing protein formin promotes axon extension. Moreover, RhoA-mDia signaling enhances axon elongation by stabilizing and assembling growth cone microtubules, whereas RhoA-ROCK signaling restricts microtubule assembly and growth cone protrusion [299]. In terms of RhoA’s role in regulating microtubule dynamics in PC12 cells, recent studies have shown that RhoA signaling pathway inhibits neurite outgrowth by increasing Glu-tubulin levels and downregulating the microtubule-severing proteins spastin and p60-ketanin in the dorsal root ganglion (DRG) and neuronally differentiated PC12 cells [300]. The C-terminal amino acid of most α-tubulin proteins is tyrosine, which can be cleaved by tubulin carboxypeptidase, exposing a glutamate residue. This detyrosinated tubulin (Glu-tubulin) is present on stable microtubules, ensuring microtubule stability [301]. Conversely, microtubules regulate RhoA activity. GEF-H1 (also known as Lfc and ARHGEF2), a RhoA GEF, remains inactive when bound to polymerized microtubules; however, it becomes active when released from depolymerized microtubules, leading to RhoA activation [302,303] (Figure 18). These findings suggest that microtubules can activate RhoA, potentially interfering with neurite outgrowth in neurons. Additionally, the primary function of collapsin response mediator protein 2 (CRMP2) is to promote growth cone advancement by regulating microtubule assembly and numb-mediated endocytosis. However, the phosphorylation of CRMP2 by ROCK leads to growth cone collapse, as phosphorylated CRMP2 (p-CRMP2) binds to actin rather than tubulin. Additionally, ephrin-A5 triggers CRMP2 phosphorylation [304] (Figure 18).

### 7.9. Relationship Between Aβ and RhoA in AD

The RhoA signaling pathway is closely associated with AD [305]. The RhoA/ROCK signaling pathway is critical for Aβ generation and deposition. ROCK2 phosphorylates APP at the Thr654 residue, a site known to be critical for APP processing to Aβ. Although ROCK2 knockdown reduces Aβ levels, it has been suggested that this reduction occurs through a mechanism independent of Thr654 phosphorylation [306]. Notably, ROCK1 activity is elevated in the frontal cortex and hippocampal neurons of both APP/PS1 mice and AD patients. ROCK1 can phosphorylate APP at Ser655, with increased levels of p-Ser655 APP observed in the brain of APP/PS1 mice and AD patients compared to controls. This phosphorylated form, p-Ser655 APP, serves as an improved substrate for BACE1, an enzyme involved in Aβ production. Consequently, ROCK1 knockdown or the use of ROCK inhibitor Y27632 reduces amyloid pathology and improves learning and memory in APP/PS1 mice [307]. ROCK1 protein levels are significantly upregulated in AD. Additionally, Aβ42 markedly increases levels of phosphorylated LIM kinase (p-LIMK, a downstream target of ROCK) in cortical primary neurons, even though the overall protein levels of ROCK1 and ROCK2 remain marginal, suggesting that Aβ42 promotes ROCK kinase activity. Conversely, the shRNA targeting of ROCK1 and ROCK2 or heterozygous ROCK1 knockout (+/−) mice show reduced Aβ42 levels via lysosomal degradation. This implies that Aβ promotes RhoA/ROCK activity, which in turn enhances Aβ generation, creating a vicious cycle in AD through positive feedback loops [308].

Aβ promotes ROS generation through the activation of the RhoA/ROCK pathway and p47phox, a cytosolic component of NADPH oxidase [126]. In the microglia, ROCK enhances the expression of nitric oxide synthase and TNF-α, contributing to neurodegeneration in response to toxic agents such as methylmercury [309]. Additionally, the Aβ-induced activation of RhoA/ROCK signaling pathway in BV2 cells is associated with microglial migration, cytotoxicity and inflammatory response. Elevated RhoA expression is observed in reactive microglia of transgenic APP/PS1 and in mice with stereotactically injected fibrillar Aβ [310]. Furthermore, ROCK inhibition in rats has been shown to block toxic M1-type microglial polarization and neuroinflammation [311]. These findings suggest that Aβ initiates a vicious cycle of neuroinflammation, contributing to neurodegeneration via the RhoA signaling pathway. In 18-month-old APP Tg2576 mice (Swedish mutation), RhoA expression decreases in synapses but increases in dystrophic neurites. In AD and Pick’s disease, RhoA colocalizes with hyperphosphorylated inclusions, likely due to sequestration by neurofibrillary tangles, whereas the localizations of Rac1, Cdc42, and p21-activated kinase (PAK) remain unchanged [312]. Early studies revealed that RhoA and its effector protein ROCK regulate Aβ42 production in vitro. The selective ROCK inhibitor Y27632 has been shown to reduce Aβ42 levels in a transgenic mouse model of AD. Notably, non-steroid anti-inflammatory drugs (NSAIDs), including sulindac sulfide, S-ibuprofen, R-ibuprofen, and indomethacin, decrease both RhoA-GTP and Aβ42 levels, suggesting that NSAIDs may inhibit RhoA activity via an unknown mechanism [313].

Synapse and dendritic spine loss induced by AβOs is a key hallmark of the early stages of AD, correlating with the cognitive decline characteristic of this condition [314]. Aβ reduces neurite outgrowth while activating RhoA, inactivating Rac1, and phosphorylating CRMP2 in the cerebral cortex of APP(Swe) Tg2576 mice. Moreover, Y27632, a Rho kinase inhibitor, reduces the threonine phosphorylation of CRMP2, suggesting that ROCK may phosphorylate CRMP2 [315]. Mammalian Ste20-like kinase 3 (Mst3), a serine/threonine kinase, is highly expressed in the developing mouse brain and is essential for radial neuronal migration, contributing to the lamination of the cerebral cortex. One mechanism involves CDK5, a serine/threonine kinase also crucial for neuronal migration, which phosphorylates Mst3 at Ser79, thereby enhancing its kinase activity. Subsequently, Mst3 phosphorylates RhoA at Ser26, potentially inactivating RhoA by disrupting its interaction with GEFs. Although the exact mechanism by which inactive RhoA facilitates neuronal migration remains largely unknown, it is hypothesized that Mst3-induced RhoA inactivation leads to F-actin depolymerization, ultimately enabling neuron polarization and their exit from the intermediate zone—a critical initial step in radial migration [316]. ROCKs can phosphorylate Tau at Thr245, Thr377, and Ser409 residues, with minor phosphorylation at Ser262 residue, and phosphorylate MAP2 at Ser1796 residue, consequently reducing their affinity for the cytoskeleton. Additionally, both Tau and MAP2 interact with the myosin-binding subunit (MBS) of myosin phosphatase. These findings indicate that Tau and MAP2 are possible substrates of ROCK and myosin phosphatase [183]. Levels of ROCK1 protein levels are elevated in the mild cognitive impairment (MCI) stages of AD. Aβ42 oligomers strongly promote the phosphorylation of LIMK1 through enhancing ROCK’s enzyme activity, while both ROCK1 and ROCK2 stimulate Aβ40 production. A hypothesis has been proposed that Aβ accumulation in MCI AD activates the RhoA/ROCK pathway, which, in turn, induces further Aβ production, creating a long-term, self-sustaining cycle that contributes to amyloid pathology in AD [308].

### 7.10. Rho GTPases in Spine Formation

**Figure 19:**
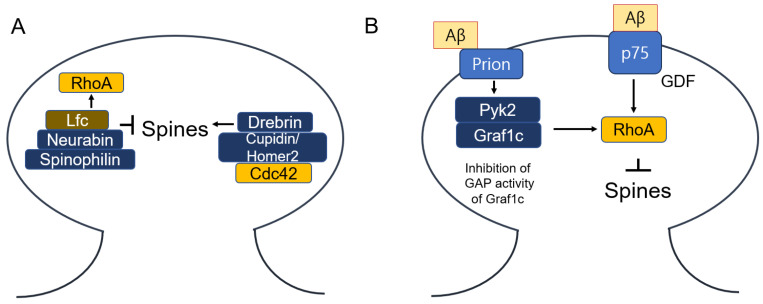
Regulation of dendritic spine formation by RhoA and Cdc42. (**A**) The complex of Lfc, neurabin, and spinophilin activates RhoA, leading to impaired dendritic spine formation. (**B**) Aβ activates Pyk1 via the prion receptor, and the association of Pyk1 with Grafi1c, a RhoGAP, inhibits Graf1c activity, resulting in RhoA activation and the disruption of dendritic spine formation.

Extensive research verifies that altering Rho GTPases activity can influence spinogenesis in developing neurons [317]. Increased activities of Rac1 and Cdc42 promote and sustain dendritic spines, while RhoA is involved in the pruning of immature spines [318]. Lfc, also known as Lbc (lymphoid blast crisis)’s first cousin)/GEF-H1/ARHGEF2, a GEF for RhoA [319], is highly expressed in the brain and interacts with neurabin and spinophilin [320]. Neurabin and spinophilin are actin-binding proteins that regulate dendritic spine formation and morphology [321]. Under basal conditions, Lfc associates with microtubules, but it translocates to spines in response to neuronal stimulation. The expression of Lcf significantly decreases spine length and area but increases spine density [320]. Interestingly, mice lacking spinophilin show a marked increase in spine density during development, and their neurons exhibit enhanced filopodial protrusion [322]. These findings indicate that Lfc, spinophilin, and possibly neuaibin may play roles in retracting spine formation or suppressing initial spine outgrowth from the dendrites. Conversely, the expression and localization of the drebrin A isoform, which accumulates in dendritic spines, support robust dendritic spine formation [323]. Drebrin interacts with Cdc42, a regulator of dendritic spines, through Cupidin/Homer2 [324] (Figure 19). Lfc is highly expressed in the embryonic brain, suggesting its importance in brain development [320]. GEF-H1 binding to microtubule reduces its activity, but when released from depolymerized microtubules, its activity increases, leading to RhoA activation [302]. Additionally, GEF-H1 activity is significantly upregulated by dephosphorylation and translocation to synaptic membranes and nuclear structures during the early reperfusion after transient cerebral ischemia [325].

Excessive synaptic loss is proposed to be one of the earliest events in AD. The acute overproduction of either axonal or dendritic Aβ reduces spine density and plasticity at nearby dendrites [326]. Soluble Aβ interacting with the cell membrane promotes RhoA activation, leading to growth cone collapse and neurite retraction in cultured hippocampal neurons. Concurrently, Aβ inhibits histone deacetylase 6 (HDAC6) activity, increasing levels of acetylated Tau and tubulin, which destabilizes microtubules and disrupts axonal integrity, as acetylated Tau is mislocalized in dendrites [327]. However, a direct link between RhoA activation and HDAC6 inactivation was not investigated in this study. A soluble Aβ oligomer at nanomolar concentration binds to p75^NTR^ in dendritic spines, activating the RhoA/ROCK signaling pathway and resulting in dendritic spine pathology [314]. AβOs also activate the intracellular tyrosine kinase Pyk2 in the dendrite spines of hippocampal neurons through receptors such as prion. Pyk2 recruits Graf1c, a RhoA GTPase-activating protein, and Pyk2 inhibits Graf1c activity, resulting in an active state of RhoA, which contributes to dendritic spine loss [328] (Figure 19). Interestingly, recently, research has shown that Aβ deposition is linked to brain network hyperexcitability, whereas p-Tau deposition mainly induces brain network hypoexcitability in transgenic models, as observed in electrophysiological studies [329].

## 8. Conclusions

Two characteristic features of AD are amyloid plaques composed of Aβ and neurofibrillary tangles consisting of hyperphosphorylated Tau proteins, which influence each other. A high concentration of Aβ is detrimental for neurons and other brain cells, either directly or through specific receptors. Specifically, Aβ oligomers trigger the microglia to induce ROS and several factors harmful to brain cells. Tau functions primarily to stabilize the microtubule, but hyperphosphorylated Tau (p-Tau) dissociates from microtubules and aggregates, leading to microtubule destabilization and impairments in axons and dendrites. Aβ-activated RhoA is closely associated with axonal retraction and dysfunction. Although inhibitors targeting ROCK, which is activated by active RhoA, have been developed as potential AD treatments, they are limited by side effects.

## 9. Perspectives

ROCK inhibitors have potential therapeutic applications across a wide range of pathological conditions, including asthma, cancer, erectile dysfunction, glaucoma, insulin resistance, kidney failure, neuronal degeneration, and osteoporosis. To date, two ROCK inhibitors have been approved for clinical use in Japan (fasudil and ripasudil), and one has been approved in China (fasudil). Fasudil was first approved in 1995 for treating cerebral vasospasm, and more recently, ripasudil was approved for glaucoma treatment (in 2014) [330]. Inhibiting ROCK activation with fasudil reduces Tau phosphorylation and promotes neurite outgrowth in hippocampal neurons following Aβ treatment [331]. Beyond decreasing Aβ deposition, Tau phosphorylation, and BACE expression, fasudil also reduces inflammation markers, including TLR-2/4, p-NF-κB/p65, MyD88, interleukin-1β, interleukin-6, and tumor necrosis factor-α, in APP/PS1 mice [332]. Among the various inhibitors developed to reduce Tau aggregation, thiazovidin is a novel ROCK-targeting compound that decreases aggregated Tau levels in N2a cells [333]. FSD-C10 inhibits p-Tau formation, Aβ expression, and β-secretase activity in the hippocampus and cortex area of APP/PS1 transgenic mice, thereby significantly improving learning and memory impairment in APP/PS1 mice [334]. Many researchers have proposed that both ROCK1 and ROCK2 could serve as therapeutic targets to reduce Aβ production in AD [308]. In 3D spine morphometry analyses, Y-27632—a pan-ROCK small molecule inhibitor—significantly increases the mean protrusion intensity while decreasing the mean protrusion width in dendritic spines. Specifically, Y27632 increases the number of filipodia and thin spines but does not affect the numbers of stubby and mushroom spines [335]. Elevated levels of insoluble Tau are associated with increased ROCK1 and ROCK2 protein levels in supranuclear palsy (PSP) and corticobasal degeneration (CBD). Both fasudil and, more effectively, the ROCK2 inhibitor SR3677 reduce p-Tau (Ser202 and Ser396) and insoluble Tau levels. Additionally, ROCK2 knockdown reduces p70 S6 kinase and phosphorylated mTOR levels, suggesting that ROCK2 inhibition enhances the autophagy of Tau [336]. The brains of AD model mice treated with fasudil via peripheral IP injection have shown the reverse gene expression seen in brains with neurodegenerative diseases such as AD [337].

The ROCK inhibitor fasudil was primarily developed as a vasodilatory drug and licensed in Japan in 1995. Researchers have conducted clinical trials of fasudil for cardiovascular disease, such as angina pectoris, Raynaud’s syndrome, pulmonary hypertension, and arterial hypertension [338]. Fasudil was tested as an early-stage amyotrophic lateral sclerosis (ALS) drug in phase IIa [339,340]. In other diseases, such as cancer, AT13148 AGC kinase inhibitor, which potentially inhibits ROCK and AKT kinases and inhibits proliferative and metastatic activity in preclinical models, was clinically tried in oral administration [341]. Very recently, a phase I trial of fasudil for oral application was conducted to assess bioavailability, safety, tolerability in healthy participants [342]. However, fasudil’s clinical application is limited by its high toxicity and narrow safety margin, restricting its use to short-term administration. Moreover, due to poor oral bioavailability, fasudil can only be administered intravenously in clinical settings [343]. Notably, the RhoA-specific inhibitor Rhosin was found to inhibit GEF-catalyzed RhoA activation without affecting the signaling activities of Cdc42 or Rac1. Moreover, Rhosin promotes neurite outgrowth in PC12 cells synergistically with NGF [344]. Rhosin also prevents both the hyperexcitability in D1-MSN (dopamine 1 receptor medium spiny neurons) and the reduced excitatory input to D1-MSNs induced by social defeat stress. Nucleus accumbens-specific RhoA inhibition can counteract the susceptibility caused by D1-MSN EGR3 (early growth response 3) expression. Additionally, Rhosin enhances spine density, which correlates with D1-MSN excitability, without affecting overall dendritic branching [345]. The bacterial enzyme C3-ADP ribosyltransferase (C3) selectively and irreversibly inhibits RhoA by ADP-ribosylation at the Asn41 residue of RhoA using NAD^+^ as a substrate [346]. C3-ADP ribosyltransferase has shown success in promoting axon regeneration in the central nervous system [347,348]. However, when C3’s effect on the regeneration of peripheral nerve injuries was examined in spinal cord nerves, C3 failed in peripheral nerve regeneration [349].

Although inhibitors targeting the RhoA/ROCK pathway show promise as a strategy for AD treatment, it is essential to develop AD-specific inhibitors to minimize serious side effects. For instance, Aβ-specific antibody-conjugated drug delivery could be an effective approach for treating AD. Additionally, inhibitors for kinases of Tau phosphorylation, including GSK-3β, CDK5, p38MAPK, c-Abl, Src, Fyn, DYRK1A, MARK4, SKY, and ROCK, have been developed. However, no inhibitors have been established to effectively improve AD [154].

## Data Availability

All experimental datasets throughout the current study are available on reasonable request to corresponding author.

## Abbreviations

4EBP: initiation factor 4E binding protein; Aβ, amyloid-β peptide; AD, Alzheimer’s disease; APP, amyloid precursor protein; AβOs, Aβ oligomers; fAβ, fibrillar Aβ; ALS, amyotrophic lateral sclerosis; AICD, APP intracellular domain; APH, anterior pharynx-defective 1; Arp2/3, actin-related protein2/3; AJ, adherens junction; ARAP3, Rap-dependent RhoGAP; BACE, β-site amyloid precursor protein cleaving enzyme, β-secretase; BDNF, brain-derived neurotrophic factor; CTF, C-terminal fragment; CK, casein kinase; CR, complement receptor; C/EBPβ, CCAAT/enhancer-binding protein β; CREB, cAMP response element-binding; CRMP2, collapsin response mediator protein 2; C3G, RapGEF1; DPI, diphynyleneiodonium; DYRK1A, dual specificity tyrosine-phosphorylation-regulated kinase 1A; GAP, GTPase-activating proteins; srGAP, Slit-Robo GAP; GDI, guanine nucleotide dissociation inhibitor; GDF, GDI displacement factor; GEF, guanine nucleotide exchange factor; GSK-3β, glycogen synthase-3β; CDK5, cyclin-dependent kinase 5; CDX, doublecortin; CaMKII, calcium/calmodulin-dependent protein kinase II; DCC, deleted in colorectal cancer; Eph receptor, erythropoietin-producing hepatocellular carcinoma receptor; FcR, Fc receptor; FPR2, formyl peptide receptor 2; JNK, C-Jun N-terminal kinase; HDAC6, histone deacetylase 6; HSPGs, heparan sulfate proteoglycans; IL, interleukin; LKB1, liver kinase B1; IKK, IκB kinase; LRP1, low-density lipoprotein receptor-related protein 1; LIMK, LIM-kinase; LPA, lysophosphatidic acid; MLCK, myosin light chain kinase; MAP2, microtubule-associated protein 2; MARCO, macrophage receptor with collagenous structure; MAG, myelin-associated glycoprotein; OMgP, myelin oligodendrocyte glycoprotein; MRCKα, myotonic dystrophy kinase-related Cdc42-binding kinase α; mGluR5, metabotropic glutamate receptor 5; MARK, microtubule-associated protein (MAP)-microtubule affinity regulating kinase (Par-1 in *C. elengans*; KIN1 in *S. cerevisiae*; MARK in mammals); nAchR, nicotinic acetylcholine receptor; NFT, neurofibrillary tangle; NOX, NADPH oxidase; Nrf, nuclear respiratory factor; NF-κB, nuclear factor kappa B; NGF, nerve growth factor; PAK, p21-activated kinase; PKA, protein kinase A; PKB, protein kinase B, Akt; PKC, protein kinase C; PKN, protein kinase N; PrP^C^, cellular prion protein; PrP^Sc^, infectious prion protein; PTP1B, protein phosphatase 1B; RAGE, receptor for advanced glycation end products; ROCK, Rho-associated protein kinase; ROS, reactive oxygen species; PS1, presenilin 1; PPARγ, peroxisome proliferator-activated receptor gamma; RXRα, retinoid X receptor alpha; Sema3, semaphorin-3A; SR, scavenger receptor; SCARA1, scavenger receptor A1; ARB1, scavenger receptor class B type 1; TNF-α, tumor necrosis factor-α; TLRs, toll-like receptor; TrkA, tropomyosin receptor kinase A; TREM2, triggering receptor expressed on myeloid cells 2; TSC, tuberous sclerosis complex; N-WASP, neuronal Wiskott–Aldrich syndrome protein; WAVE, WASP family verprolin-homologous protein.
